# Using Cu‐Based Metal–Organic Framework as a Comprehensive and Powerful Antioxidant Nanozyme for Efficient Osteoarthritis Treatment

**DOI:** 10.1002/advs.202307798

**Published:** 2024-01-26

**Authors:** Bo Yu, Wei Sun, Juntao Lin, Chaoyu Fan, Chengxinqiao Wang, Zhisen Zhang, Yupeng Wang, Yonghua Tang, Youhui Lin, Dongfang Zhou

**Affiliations:** ^1^ Department of Orthopaedics and Traumatology & Department of Ultrasonic Diagnosis, Zhujiang Hospital Key Laboratory of Mental Health of the Ministry of Education NMPA Key Laboratory for Research and Evaluation of Drug Metabolism & Guangdong Provincial Key Laboratory of New Drug Screening, School of Pharmaceutical Sciences Southern Medical University Guangzhou 510515 P. R. China; ^2^ Department of Physics, Research Institute for Biomimetics and Soft Matter, Fujian Provincial Key Laboratory for Soft Functional Materials Research Xiamen University Xiamen 361005 P. R. China

**Keywords:** antioxidant activity, metal–organic framework, nanozyme, osteoarthritis, pro‐oxidant activity

## Abstract

Developing nanozymes with effective reactive oxygen species (ROS) scavenging ability is a promising approach for osteoarthritis (OA) treatment. Nonetheless, numerous nanozymes lie in their relatively low antioxidant activity. In certain circumstances, some of these nanozymes may even instigate ROS production to cause side effects. To address these challenges, a copper‐based metal–organic framework (Cu MOF) nanozyme is designed and applied for OA treatment. Cu MOF exhibits comprehensive and powerful activities (i.e., SOD‐like, CAT‐like, and •OH scavenging activities) while negligible pro‐oxidant activities (POD‐ and OXD‐like activities). Collectively, Cu MOF nanozyme is more effective at scavenging various types of ROS than other Cu‐based antioxidants, such as commercial CuO and Cu single‐atom nanozyme. Density functional theory calculations also confirm the origin of its outstanding enzyme‐like activities. In vitro and in vivo results demonstrate that Cu MOF nanozyme exhibits an excellent ability to decrease intracellular ROS levels and relieve hypoxic microenvironment of synovial macrophages. As a result, Cu MOF nanozyme can modulate the polarization of macrophages from pro‐inflammatory M1 to anti‐inflammatory M2 subtype, and inhibit the degradation of cartilage matrix for efficient OA treatment. The excellent biocompatibility and protective properties of Cu MOF nanozyme make it a valuable asset in treating ROS‐related ailments beyond OA.

## Graphical abstract

A Cu‐based metal–organic framework (Cu MOF) nanozyme fabricated through a simple self‐assembly strategy exhibits comprehensive and powerful antioxidant activities while minimal pro‐oxidant activities. The Cu MOF nanozyme shows excellent scavenging capabilities against various types of ROS and relieves the hypoxic microenvironment of synovial macrophages, resulting in efficient and safe treatment of osteoarthritis (OA).

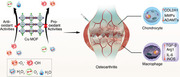

## 1 Introduction

Osteoarthritis (OA) is a prevalent and disabling condition that imposes a growing burden on patients, the healthcare system, and society at large.^[^ ^1^ ^]^ Currently, an estimated 250 million people worldwide are affected by this condition, making it increasingly burdensome.^[^ ^2^ ^]^ OA is characterized by elevated levels of a variety of reactive oxygen species (ROS), such as •O_2_^−^, •OH, and H_2_O_2_, contributing to its oxidative microenvironment and pathological changes.^[^ ^3^ ^]^ The overexpression of ROS in the inflamed joint leads to degradation of the extracellular cartilage matrix and synovitis.^[^ ^4^ ^]^ Moreover, the proliferation of synovial cells exacerbates the hypoxia of the synovium.^[^ ^5^ ^]^ Hypoxia disrupts the mitochondrial metabolism of synovial macrophages, increasing ROS production and an enhanced inflammatory response.^[^ ^6^ ^]^ Therefore, the effectiveness of OA treatment hinges on eliminating various types of ROS and mitigating the hypoxic microenvironment.^[^ ^7^, ^8^ ^]^

Natural antioxidant enzymes, such as superoxide dismutase (SOD), are part of the antioxidant system in humans.^[^ ^9^ ^]^ The direct injection of SOD has been proven a feasible strategy for treating OA.^[^ ^9^ ^]^ However, natural SOD is susceptible to inactivation and rapid metabolism within the human body. Different strategies for improving natural enzymes’ reliability, stability, and longevity by incorporating enzymes into nanomaterials have been developed for OA mitigation.^[^ ^10^ ^]^ Although promising, these as‐prepared nanocomposites cannot concurrently remove diverse types of ROS. To offer comprehensive antioxidant protection in treating inflammatory diseases, nanozymes, with their multiple antioxidant activities, have attracted researchers attention.^[^ ^8^, ^10^, ^11^ ^]^ Nevertheless, the catalytic efficiency of conventional nanozymes, such as carbon‐, metal‐, and metal oxide‐based nanozymes, remains a significant challenge due to the substantial disparity in the metal‐nitrogen active centers compared to natural antioxidant enzymes.^[^ ^8^, ^12^ ^]^ To mimic the catalytic center of natural enzymes, single‐atom nanozymes with metal‐nitrogen coordination structures have been developed recently.^[^ ^11a,c,d^ ^]^ It has been reported that single‐atom nanozymes have strong catalase (CAT)‐like activity (H_2_O_2_→O_2_+H_2_O) for antioxidant therapy and oxygen supply.^[^ ^11d^ ^]^ Unfortunately, they are usually incapable of neutralizing the highly oxidative •OH in the inflammatory microenvironment of OA. Even worse, many single‐atom nanozymes also possess high peroxidase (POD)‐ and oxidase (OXD)‐like activities, which decompose hydrogen peroxide and oxygen into highly toxic •OH^[^ ^11c^ ^]^ and •O_2_^−^,^[^ ^13^ ^]^ respectively. Inevitably, these pro‐oxidant activities may exacerbate oxidative stress damage in OA and potentially cause side effects.^[^ ^14^ ^]^ Therefore, it is crucial to design a nanozyme with comprehensive and powerful antioxidant activities while avoiding pro‐oxidant activities for safe and efficient OA treatment.

**Scheme 1:**
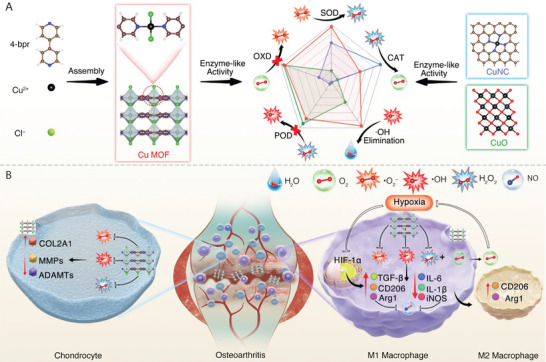
Cu MOF as a comprehensive and powerful antioxidant nanozyme for efficient osteoarthritis treatment. A) Scheme of the synthesis of Cu MOF nanozyme, and its enzyme‐like activities compared with the other two Cu‐based nanozymes (CuNC, CuO). B) Scheme of Cu MOF nanozyme for efficient osteoarthritis treatment through promoting the polarization of macrophages to M2 subtype by clearing excess intracellular ROS and improving hypoxia, thus inhibiting synovitis and cartilage degeneration.

Metal–organic frameworks (MOFs) with diverse metal nodes and organic linkers have been developed as promising candidates for artificial enzymes.^[^ ^15^ ^]^ Herein, we present a straightforward self‐assembly strategy for fabricating a copper‐based metal–organic framework (Cu MOF) nanozyme with comprehensive and powerful activities (i.e., SOD‐like, CAT‐like, and •OH scavenging activities) while almost negligible pro‐oxidant activities (POD‐ and OXD‐like activities) for OA therapy. Density functional theory (DFT) also confirmed the excellent enzyme activities of Cu MOF. Therefore, the as‐obtained Cu MOF nanozyme provides efficient scavenging capabilities against various types of ROS (**Scheme** **1**A). Simultaneously, with its efficient oxygen production capacity, Cu MOF nanozyme mitigates the hypoxic microenvironment of OA synovial macrophages. Under the combined action, it promotes the transition of macrophages from the M1 phenotype to the M2 phenotype, thereby reducing the secretion of pro‐inflammatory factors. Furthermore, it inhibits cartilage degeneration by improving the highly oxidative microenvironment (Scheme 1B). This study provides a fresh perspective on designing highly efficient antioxidant nanozymes and their application in OA treatment.

## 2 Results and Discussions

### 2.1 Synthesis and Characterization of Cu MOF

**Figure 1:**
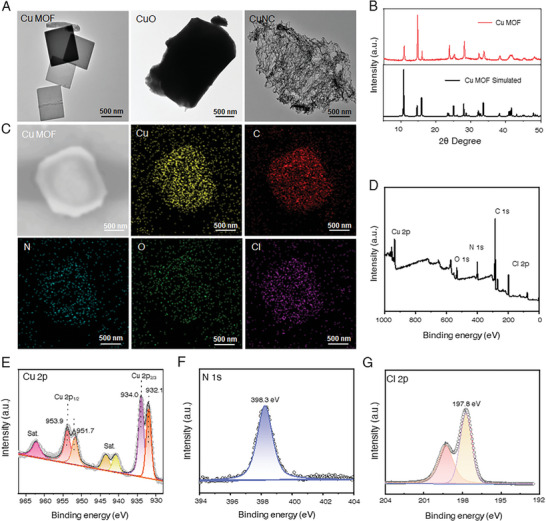
Morphology and structure characterizations of Cu MOF. A) TEM images of Cu MOF, CuO, and CuNC; B) XRD patterns of Cu MOF; C) EDS elemental mappings of Cu MOF; D–G) XPS spectra of Cu MOF: D) survey spectrum, E) Cu 2p spectrum, F) N 1s spectrum, G) Cl 2p spectrum.

Highly active and homogeneous CuNx sites were anchored on porous Cu MOF by combining Cu atoms and interconnected N (4,4′‐bipyridine) using a self‐assembly technique,^[^ ^16^ ^]^ as shown in Scheme 1A. The morphology of the Cu MOF was observed by using transmission electron microscopy (TEM) and scanning electron microscope (SEM). **Figures** **1**A and S1 (Supporting Information) showed regular cubic blocks of Cu MOF ≈1.5 µm. The UV–vis spectrum reveals a redshift of 6.76 nm in the peak of 4,4′‐bipyridine at 388.48 nm upon self‐assembly with Cu ions. However, the shape of the peak remained unchanged (Figure S2, Supporting Information). This suggests that 4,4′‐bipyridine remained unaltered within the Cu MOF, indicating the successful construction of the CuNx site. X‐ray diffraction (XRD) analysis further supported the presence of the constructed CuNx sites in the MOFs, as the pattern obtained from the prepared Cu MOF matched the simulated XRD pattern. This finding was further supported by energy dispersive spectroscopy (EDS) mapping analysis, which demonstrated a uniform distribution of Cu and N elements on the surface (Figure 1C), affirming the generation of CuNx sites within the 3D matrix. Notably, the oxygen element in the MOF structure may come from the water of crystallization of the structure and oxygenated species adsorbed on the surface. As evidenced by the thermogravimetric data at temperatures higher than 100 °C, the mass of the Cu MOF begins to decrease slowly (from the water) (Figure S3A, Supporting Information). Nitrogen adsorption isotherm measurements and the corresponding pore size distribution analyses (Figure S3B,C, Supporting Information) indicated that the Cu MOF possesses a porous structure with an average pore size of ≈1.5 nm. This porous structure was advantageous for exposing the Cu active sites.^[^ ^17^ ^]^

**Figure 2:**
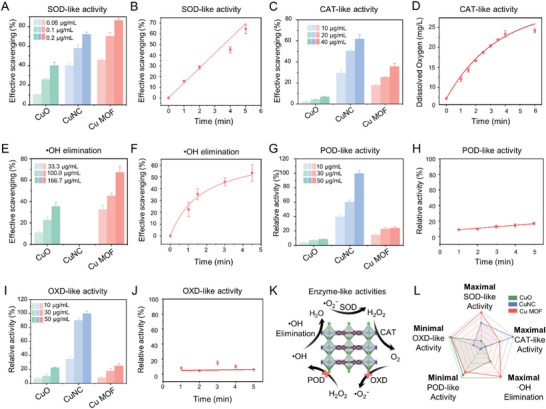
Comprehensive and powerful antioxidant and negligible pro‐oxidant activities of Cu MOF nanozyme. A) SOD‐like activity of different Cu‐based nanozymes at different concentrations; B) SOD‐like activity versus time curves of Cu MOF nanozyme; C) CAT‐like activity of different Cu‐based nanozymes at different concentrations; D) CAT‐like activity versus time curves of Cu MOF nanozyme; E) Elimination of •OH activity of different Cu‐based nanozymes at different concentrations; F) Elimination of •OH activity versus time curves of Cu MOF nanozyme; G) POD‐like activity of different Cu‐based nanozymes at different concentrations. H) POD‐like activity versus time curves of Cu MOF nanozyme; I) OXD‐like activity of different Cu‐based nanozymes at different concentrations; J) OXD‐like activity versus time curves of Cu MOF nanozyme; K) Schematic diagram of the antioxidant and pro‐oxidant activities of Cu MOF nanozyme; L) Radar graph for Summary of different nanozymes indicators of enzyme‐like activities.

The binding states of Cu, C, N, O, and Cl on Cu MOF were further investigated by using X‐ray photoelectron spectroscopy (XPS) (Figures 1D–G; S4, Supporting Information). The deconvolution of Cu 2p, as shown in Figure 1E, revealed two prominent peaks at 932.5 and 934.4 eV, respectively, which could be well assigned to 2p_3/2_ of Cu^+^ and Cu^2+^ species.^[^ ^18^ ^]^ Figure 1F exhibited a distinct peak centered at 398.5 eV, indicative of the N species associated with the N─Cu bond. This result suggested that pyridine N species serve as stable anchor points for Cu atoms, akin to conventional metal–N–C catalysts. According to **Figure** **2**G, the Cl 2p peaks at 197.8 eV indicate the presence of Cu–Cl species.^[^ ^19^ ^]^ In addition, XPS displayed that the elemental content of Cl (9.12%) and N (11.16%) is ≈1.6 and 1.9 times of Cu (5.84%) (Table S1, Supporting Information), which was consistent with the Cl_2_–Cu–N_2_ coordination in Cu MOF crystal structure, respectively.

To elucidate the function of the CuN (CuN_2_) active site in the simulated Cu MOF, two control samples were employed, namely, CuO containing the Cu–O site and a Cu single‐atom nanozyme with Cu–N (CuNC). The morphology and structure of two control samples were also characterized. The TEM images show that the commercial CuO is a smooth‐surfaced cubic bulk of ≈1.5 µm (Figure 1A). XRD further confirmed the crystal phase of CuO spheres (Figure S5, Supporting Information). CuNC was synthesized using a two‐step annealing method, following previously reported procedures.^[^ ^20^ ^]^ The magnified TEM image displayed an absence of particles, indicating the dispersion of Cu in a monatomic Cu–N coordination structure on the carbon carrier (Figure 1A, Figure S6A, Supporting Information). This observation was corroborated by XRD measurements, where the spectrum exhibited only a broad carbon peak near 27°,^[^ ^20^ ^]^ without any peaks of Cu particles (Figure S6B, Supporting Information). CuO and CuNC were reported to have SOD‐ and CAT‐like activities,^[^ ^21^ ^]^ but very few reports on •OH removal activity. Besides that, they tend to have high POD‐ or OXD‐like activity, which severely limits their application in anti‐inflammation therapy.^[^ ^22^ ^]^

### 2.2 Comprehensive and Powerful Antioxidant and Negligible Pro‐Oxidant Activities of Cu MOF Nanozyme

Three representative ROS, including H_2_O_2_, •O_2_^−^ and •OH, were selected to investigate the ROS scavenging activities of the three different Cu‐based nanozymes. The SOD‐like activity of different Cu‐based nanozymes was initially assessed (Figure 2A; Figure S7, Supporting Information). As shown in Figure 2A, ≈84% of the •O_2_^−^ was decomposed when treated with 0.2 µg mL^−1^ Cu MOF, representing a 1.5 and 4‐fold increase compared to CuNC and CuO, respectively. Moreover, compared to widely used SOD‐simulated CeO_2_ and PB nanozymes (Table S2, Supporting Information), the concentration of Cu MOF required for the same •O_2_^−^ removal efficiency was reduced by 2 orders of magnitude. Similar to other nanozymes, the SOD‐like activity of Cu MOF exhibited dose‐ and time‐dependent behavior (Figure 2A,B).

Subsequently, the CAT‐like activity of Cu MOF, responsible for H_2_O_2_ decomposition, was studied. As shown in Figure 2C, the H_2_O_2_ consumption detected by fluorescence spectroscopy confirmed that the H_2_O_2_ catalytic activity of Cu MOF was ≈60% of CuNC, but much higher than that of commercial CuO (Figure S8, Supporting Information). The CAT‐like activity of Cu MOF was further demonstrated by the catalytic formation of O_2_ bubbles upon adding Cu MOF to the H_2_O_2_ solution. The O_2_ concentration in the H_2_O_2_ solution containing Cu MOF increased rapidly within 5 min (Figure 2D), consistent with the results of the bubble experiment (Figure S9, Supporting Information).

Crucially, •OH is the most oxidizing ROS in the human body, particularly in OA. The catalytic activity of •OH is a crucial factor affecting the anti‐inflammatory efficacy of nanozymes. The •OH removal activity of Cu MOF assessed using by UV–vis absorption spectroscopy with salicylic acid as a probe in a Fenton reaction system (Fe^2+^/H_2_O_2_) (Figure S10, Supporting Information). Figure 2E,F shows that the absorption intensity significantly decreased with adding Cu MOF, confirming its •OH scavenging activity. Eliminating •OH increased with higher Cu MOF concentrations and prolonged treatment times. At the concentration of 33.33 µg mL^−1^, the •OH scavenging activity of Cu MOF was 3.3 times higher than that of CuO (Figure 2E), achieving ≈84% elimination with 333.3 µg mL^−1^ Cu MOF (Figure S10, Supporting Information).

In particular, although single‐atom nanozyme CuNC performed well in CAT‐like activity, but did not eliminate •OH (Figure 2E). Conversely, CuNC exhibited POD‐ or OXD‐like activity, which produces highly toxic ROS under acidic conditions (OA microenvironment and intracellular microenvironment). For example, CuNC can catalyze the decomposition of H_2_O_2_ to generate •OH under pH 5, demonstrating remarkable POD‐like activity (Figures 2G,H; S11, Supporting Information). This may also be the reason why it is inefficient in •OH removal experiments. The OXD‐like activity of CuNC is also much higher than that of Cu MOF and CuO, which could catalyze O_2_ to •O_2_^−^ (Figures 2I,J; S12, Supporting Information). The CuNC nanozyme may be absorbed by normal tissues during antioxidant treatment in vivo, resulting in highly toxic ROS that can damage cells. In contrast, Cu MOF nanozyme exhibits comprehensive and efficient antioxidant activities (SOD, CAT, and •OH elimination), especially SOD, while its pro‐oxidant activities (OXD and POD) were negligible avoiding potential side effects (Figure 2K). From the integration of the above results, although CuO exhibits minimal pro‐oxidant activity, its antioxidant capacity was relatively weak. On the other hand, CuNC demonstrates favorable SOD‐ and CAT‐like activities but cannot scavenge •OH. Notably, CuNC exhibits robust POD and OXD‐like activities. Therefore, among these Cu‐based nanozymes, Cu MOF shows the most significant potential for safe and effective OA treatment (Figure 2L).

### 2.3 Theoretical Evaluation of Efficient Antioxidant and Negligible Pro‐Oxidant Activities of Cu MOF Nanozyme

**Figure 3:**
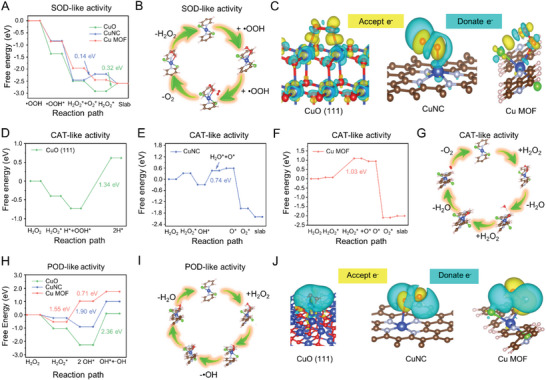
DFT theoretical evaluation of efficient antioxidant and negligible pro‐oxidant activities of Cu MOF nanozyme. A) The energy profiles correspond to the SOD‐like activity mechanism in the Cu site on the surface of CuO(111), CuNC, and Cu MOF. “^*^” represents the nanozyme surface. B) Proposed reaction process for SOD‐like activity of Cu MOF; C) Calculated charge density differences of •OOH adsorbed on the surfaces of CuO(111), CuNC, and Cu MOF. Yellow and light green regions indicate electron accumulation and electron depletion, respectively. D–F) The energy profiles corresponding to the CAT‐like activity mechanism in the Cu site on the surfaces of CuO(111), CuNC, and Cu MOF. G) Proposed reaction process for CAT‐like activity of Cu MOF. H) The energy profiles corresponding to the POD‐like activity mechanism in the Cu site on the surfaces of CuO(111), CuNC, and Cu MOF. I) Proposed reaction process for POD‐like activity of Cu MOF. J) Calculated charge density differences of H_2_O_2_ adsorbed on the surfaces of CuO(111), CuNC, and Cu MOF.

The excellent antioxidant activities of Cu MOF may result from a metal active center similar to that of natural enzymes (Figure S13, Supporting Information).^[^ ^23^ ^]^ Density function theory (DFT) was employed to analyze the reaction pathways and crucial intermediates for enzyme‐like activities, further exploring the structure‐activity relationship. All calculations are based on the three models in Figure S14 (Supporting Information). First, DFT calculations were performed on the disproportionation process of the characteristic substrate molecule of SOD, •OOH (a protonated form of •O_2_^−^),^[^ ^12^ ^]^ at different Cu sites. As shown in Figure S15 (Supporting Information), the Cu in Cu MOF loses the fewest electrons compared to the Cu sites in CuO (111) and CuNC, favoring more electrons for subsequent disproportionation processes. The analysis of the *d*‐band centers of the Cu site in the projected density of states further supports this result. The *d*‐band centers of the Cu sites are −5.3, −3.2, and −3.0 eV in the CuO (111), CuNC, and Cu MOF models, respectively (Figure S16, Supporting Information). The closer one gets to the Fermi energy level; the more electrons will likely be involved in the subsequent catalytic process. The possible SOD‐like reaction mechanisms are illustrated in **Figure** **3**A,B. The whole process of •OOH evolution on the Cu MOF surface is thermodynamically favorable, while the rate‐determining step energy of CuNC was 0.14 eV and that of CuO was 0.32 eV. Thus, Cu MOF has the best SOD‐like activity, followed by CuNC, and the weakest is CuO, which agrees with the results obtained experimentally.

To further understand the origin of the Cu MOF nanozyme activity, the electronic structure data associated with the •OOH adsorbed on the surface of Cu MOF were also calculated. As shown in Figure S17 (Supporting Information), the •O_2_^−^ molecule was first adsorbed to the CuO (111), CuNC, and Cu MOF surfaces with high chemisorption energy of −1.34, −0.83, and −0.85 eV, respectively, suggesting the steady adsorption of •O_2_^−^ on the catalytic sites. This was further demonstrated by the charge density difference of •OOH on the CuO (111), CuNC, and Cu MOF surfaces (Figure 3C). After adsorption of •OOH, electron charge donation (light blue) is observed on all three surfaces, and electron charge accumulates between •OOH^*^ and the surface (yellow), forming bonds. Therefore, the excellent SOD‐like activity of Cu MOF is attributed to two factors: the interaction between Cl and •OOH, and the interaction of the Cu site with •OOH.

Comparing the free energy path results of DFT (Figure 3D–G; Figure S18, Supporting Information), the reaction paths of CAT‐like reaction for there three Cu‐based nanozymes are different, where H_2_O_2_^*^→H_2_O^*^+O^*^, H_2_O_2_^*^→H_2_O^*^+O^*^ and H^*^+OOH^*^→2H^*^ are the decisive velocity steps for Cu MOF, CuNC and CuO, respectively. The energy barriers are 1.03, 0.74, and 1.34 eV. Therefore, the order of their CAT‐like activity was CuNC > Cu MOF > CuO, consistent with the experimental results (Figure 2D). H_2_O_2_, as a reactant for CAT‐ and POD‐like processes, and its differential charge density profiles on the surface of different nanozymes were similarly calculated. Significant charge transfer between the surface of different nanozymes and H_2_O_2_ can be seen in Figure 3J.

DFT was similarly used to calculate the evolutionary pathways and vital intermediates of H_2_O_2_ on their surfaces to compare their POD‐like activities. Figure 3H,I shows that the rate‐determining step for CuNC and CuO is the dissociation of the •OH, which requires crossing energy barriers of 1.9 and 2.36 eV, respectively. However, the rate‐determining step for Cu MOF is indeed H_2_O_2_^*^→2OH^*^ with an energy barrier of 1.55 eV, and subsequent •OH desorption still requires crossing another energy barrier of 0.71 eV. Therefore, the reaction order of their POD‐like activities is CuNC > Cu MOF > CuO, which is also consistent with the results of our experimental measurements. Based on the DFT simulations, it has also been confirmed that Cu MOF exhibits comprehensive and efficient antioxidant and low pro‐oxidant activities compared to the other two Cu‐based nanozymes.

### 2.4 ROS Scavenging Ability, Hypoxia Relief Effect, and Therapeutic Efficacy of Cu MOF Nanozyme In Vitro

Based on previous conclusions and analyses, Cu MOF nanozyme exhibits a comprehensive and efficient scavenging effect on various ROS (especially •O_2_^−^ and •OH) compared to other Cu‐based nanozymes. Its enzyme‐like activities also surpass those of some reported nanozymes (Table S2, Supporting Information). Importantly, Cu MOF exhibits minimal pro‐oxidant activity and does not generate highly toxic ROS that could harm healthy tissues. Moreover, Cu MOF can produce oxygen in a controlled environment through CAT‐like activity. Given these characteristics, Cu MOF nanozyme shows promise as an effective and safe therapy for OA by addressing ROS and the hypoxic microenvironment.

**Figure 4:**
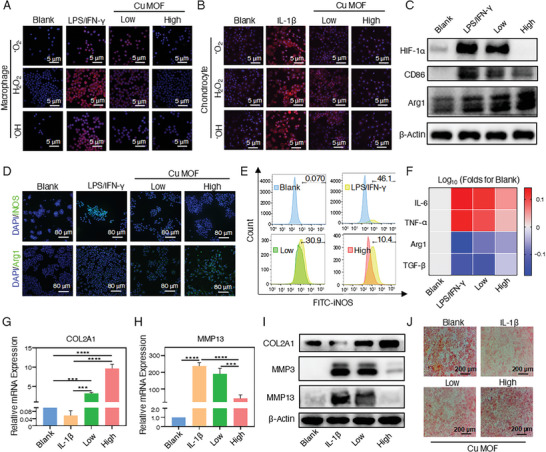
Figure 4. ROS scavenging ability, hypoxia relief effect, and therapeutic efficacy of Cu MOF nanozyme in vitro. CLSM detected the •O_2_^−^, H_2_O_2,_ and •OH of A) macrophages and B) chondrocytes treated with Cu MOF. Three types of ROS and nucleus were stained with red fluorescence and DAPI, respectively; C) Western Blotting of HIF‐1*α*, CD86, and Arg1 in macrophages treated with Cu MOF under hypoxic condition; D) Immunofluorescence staining of the macrophage markers iNOS and Arg1. Blue: DAPI, green: iNOS, Arg1; E) Flow cytometry detected the expression of iNOS in macrophage; F) ELISA analyzed the concentration of (IL‐6, TNF‐*α*, Arg1, and TGF‐*β*). The longitudinal axis represents the logarithm of the ratio of the concentration of inflammatory factors in each group to the Blank group; G,H) The qPCR detected the cartilage ECM metabolism markers (COL2A1 and MMP13) in IL‐1*β*‐stimulated chondrocytes treated with Cu MOF; I) Western Blotting analysis of the COL2A1, MMP3, and MMP13 in chondrocytes treated with Cu MOF; J) The safranin O staining of chondrocytes treated with Cu MOF. Data are presented as the means ± SD (n = 3). One‐way ANOVA with Tukey's multiple comparisons test was used to compare the means of the values of groups. ^*^ indicate significant differences between groups, respectively (*p* < 0.05).

The survival of macrophages and chondrocytes was almost un‐effected when the concentration of Cu MOF was 80 µg mL^−1^ in CCK‐8 experiment, showing the low cytotoxicity and biocompatibility of Cu MOF nanozyme (Figures S19 and S20, Supporting Information). Therefore, two distinct concentrations (8 and 80 µg mL^−1^) of the Cu MOF nanozyme were chosen for subsequent in vitro and in vivo experiments. To evaluate the ROS scavenging ability of Cu MOF nanozyme in vitro, different fluorescence probes of •O_2_^−^, •OH, and H_2_O_2_ were incubated with Cu MOF‐treated macrophages and chondrocytes and further imaged by CLSM. As shown in **Figure** **4**A,B, the low concentration of Cu MOF (8 µg mL^−1^) decreased all the red fluorescence intensities representing three type of ROS levels in cells, and this phenomenon was more evident at a higher concentration (80 µg mL^−1^). This result was also confirmed by flow cytometry analysis of intracellular DCFH‐DA (Figures S21 and S22, Supporting Information). The above results show that Cu MOF nanozyme exhibited an excellent scavenging ability on different types of ROS in macrophages and chondrocytes. Moreover, to verify the ability of Cu MOF nanozyme in improving the hypoxia of macrophages, the expression of hypoxia‐inducible factor‐1*α* (HIF‐1*α*) was detected by western blotting. As shown in Figure 4C, the expression of HIF‐1*α* protein in LPS/IFN‐*γ*‐activated macrophages (M1 macrophages) decreased by 88% after Cu MOF nanozyme treatment at the high concentration (Figure S24A, Supporting Information).

After scavenging ROS and relieving hypoxia, the impact of Cu MOF nanozyme on synovial inflammation and cartilage degeneration was assessed in vitro. The inhibitory effect of Cu MOF on macrophage inflammation in the hypoxic condition was also investigated by western blotting. As shown in Figure 4C, the expression of CD86 protein (M1 macrophage marker) in M1 macrophages decreased by 80% after the high‐concentration Cu MOF treatment (Figure S24B, Supporting Information). On the contrary, the expression of Arg1 (M2 macrophage marker) with anti‐inflammatory effect increased by 1.8‐fold (Figure S24C, Supporting Information). The immunofluorescence results further proved the macrophage polarization from M1 subtype to M2 subtype induced by Cu MOF nanozyme. After Cu MOF treatment, the fluorescence intensity of iNOS was reduced while the CD206 was enhanced in macrophages significantly (Figure 4D; Figure S25, Supporting Information). The mean fluorescence intensity of iNOS by flow cytometry was only 38% of the M1 macrophages in the low‐concentration group, and this level even decreased to 21% in the high‐concentration group (Figure 4E; Figure S26B, Supporting Information). The qPCR experiment also shows the same result as above (Figure S26A, Supporting Information). The above results suggest that Cu MOF nanozyme can promote the polarization of macrophages from activated M1 subtype to the anti‐inflammatory M2 subtype significantly by the improvement of hypoxia and clearance of ROS. Moreover, the changes in related inflammatory factors of macrophages were detected by ELISA (Figure 4F). The levels of pro‐inflammatory IL‐6 and TNF‐*α* in the high‐concentration Cu MOF‐treated M1 macrophages were restored to the expected levels of the blank group (M0 macrophages), consistent with the results of qPCR detection (Figure S27, Supporting Information). Meanwhile, the trends of anti‐inflammatory Arg1 and TGF‐*β* were increased as expected. Interestingly, Cu MOF treatment also reduced the nitric oxide (NO) levels in M1 macrophages with a concentration dependence (Figure S23, Supporting Information). NO is a pro‐inflammatory factor, and M1 macrophages would induce a strong inflammatory and immune response by producing NO.^[^ ^24^ ^]^ These results suggest that the inhibitory effect of Cu MOF on macrophage inflammation is achieved through its ability to scavenge ROS and relieve hypoxia effectively, thereby promoting macrophage polarization from M1 to M2 subtype and regulating the expression of related factors.

Chondrocytes are the only cells in articular cartilage, and the metabolic state of chondrocytes determines the composition change of the cartilage matrix.^[^ ^1^ ^]^ After scavenging the excess ROS in chondrocytes (Figure 4B), the levels of synthesizing and catabolic enzymes were further assessed to evaluate the effect of the Cu MOF on cartilage degeneration in vitro. The mRNA expression of COL2A1 in chondrocytes after treating with the high and low concentrations of Cu MOF nanozyme was 9.7 and 3.3‐fold higher than that of the control group, respectively (Figure 4G). Meanwhile, the expression of MMP13 in the high‐concentration Cu MOF‐treated group was only 18% of that in the IL‐1*β*‐stimulated group (positive control), which was further supported by the qPCR results of other metabolic enzymes (MMP3, ADAMTs‐4, ADAMTs‐5) (Figure 4H; Figure S28A, Supporting Information). Western blotting and immunofluorescence analysis also showed that Cu MOF nanozyme could down‐regulate the expression of MMPs (MMP3, MMP13) and up‐regulate the expression of COL2A1 in chondrocytes induced by IL‐1*β* (Figure 4I; Figures S28 and S29, Supporting Information).

Furthermore, safranin O staining was employed to assess the secretion of matrix glycosaminoglycan in primary chondrocytes. Following treatment with Cu MOF nanozyme, the staining intensity intensified gradually with increased concentration, indicating a substantial increase in the extracellular matrix glycosaminoglycan content (Figure 4J). All the above results suggest that Cu MOF nanozyme can modulate the polarization of macrophages, shifting them from pro‐inflammatory M1 to anti‐inflammatory M2 subtype, and inhibiting the degradation of cartilage extracellular matrix.

### 2.5 Inhibition of Cartilage Degeneration in CIOA Mice After Cu MOF Nanozyme Treatment

**Figure 5:**
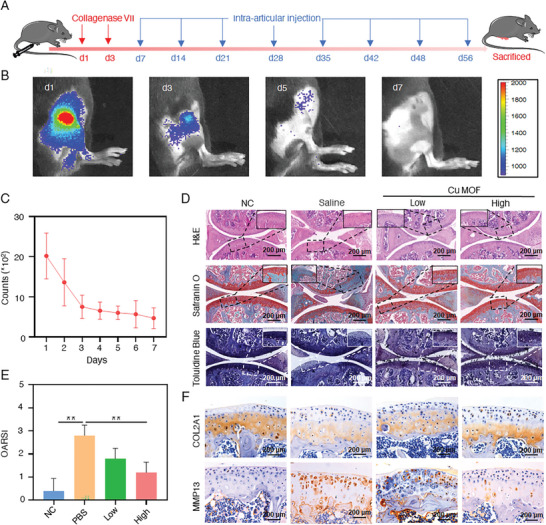
Inhibition of cartilage degeneration in CIOA mice after Cu MOF nanozyme treatment. A) Experimental process design of animal modeling and intra‐articular injection; B) IVIS images of the accumulation and C) fluorescence intensity counts of Cu MOF_IR780_ in the joints after intra‐articular injection; D) H&E staining, Safranin O staining and Toluidine Blue staining of the mice's knee region at the end of treatment; E) The OARSI scoring system evaluated the relative quantification of OA progression at the end of treatment; (E) Immunohistochemical staining of COL2A1 and MMP13 in the mice's knee region at the end of treatment. One‐way ANOVA with Tukey's multiple comparisons test was used to compare the means of the values of groups. Data are presented as the means ± SD (n = 5), ^*^ indicate significant differences compared to each groups, respectively (*p* < 0.05). Group meaning: NC (Natural control or sham operation), Saline (intra‐articular injection of saline after CIOA modeling), Low (intra‐articular injection of 8 µg mL^−1^ Cu MOF after CIOA modeling), High (intra‐articular injection of 80 µg mL^−1^ Cu MOF after CIOA modeling).

OA with high inflammatory response is a common type of knee OA disease in clinic.^[^ ^25^ ^]^ The collagenase‐induced OA (CIOA), a high synovitis‐subtype OA model, was selected to investigate the therapeutic effect of the Cu MOF nanozyme in vivo.^[^ ^26^ ^]^ As shown in **Figure** **5**A, the CIOA model is a highly inflammatory OA model in which collagen degradation in articular cartilage is induced by injection of collagenase VII, thereby causing reactive inflammation in the synovium.^[^ ^27^ ^]^ First, to determine the appropriate period and mode of administration of Cu MOF nanozyme in vivo, the accumulation period of Cu MOF in the joint after intra‐articular injection was studied using IVIS Lumina III imaging system. Cu MOF labeled with near‐infrared fluorescent dye IR780 (Cu MOF_IR780_) was synthesized, and the local fluorescence accumulation in the joint was continuously detected after the injection of Cu MOF_IR780_. As shown in Figure 5B,C, the fluorescence intensity on the second and third days decreased to 76% and 39% of the first day, respectively, and the local fluorescence intensity was shallow after 7 days. Thus, an articular injection of Cu MOF nanozyme every 7 days was administrated as a reasonable dosing cycle until 8 weeks.

At the end of the treatment, the mice's knee region was sliced for H&E, safranin O, and toluidine blue staining analyses. Compared with the NC group (normal mice), the cartilage surface of the saline group (CIOA model mice) was rougher and thinner while synovial tissue infiltration and degraded cartilage matrix were observed on the cartilage surface (Figure 5D). After Cu MOF nanozyme treatment, the H&E staining results showed that the smoothness of cartilage was improved observably, and the inflammatory infiltration damage of cartilage was also decreased. The safranin O and toluidine blue stainings also showed notable thickness improvement and matrix restoration of cartilage. The above results demonstrated the apparent recovery of cartilage matrix composition after Cu MOF nanozyme treatment. Especially, the efficacy of high‐dose Cu MOF treatment was better than that of the low‐dose group, even recovered to the same level as the NC group (Figure 5D). These results are likewise confirmed by the International Association for the Study of Osteoarthritis (OARSI) scores, in which the score in high‐dose Cu MOF group was comparable to the NC group, further indicating a obvious (though not wholly) recovery of cartilage matrix (Figure 5E).

In addition, the immunohistochemical staining of knee sections was performed (Figure 5F). The staining area and depth of COL2A1 were restored after Cu MOF treatment. At the same time, the positive ratio of MMP13 in the Cu MOF groups became smaller and lighter, indicating the reduced expression of MMP13. The efficacy of the low‐concentration group was slightly inferior to that of the high‐concentration group. The semi‐quantitative analysis of the immunohistochemical staining results showed similar results (Figure S30, Supporting Information). Therefore, Cu MOF nanozyme with comprehensive and high‐potency antioxidant activities could promote COL2A1 expression and down‐regulate MMP13 expression, thereby reducing cartilage degradation in vivo. Moreover, the in vivo biosafety of Cu MOF nanozyme was evaluated. Similar to the saline group, no histopathological abnormalities or damage to the mice organs after Cu MOF treatment (Figure S31, Supporting Information) were observed, which may be related to the low off‐target toxicity caused by the negligible pro‐oxidant enzyme activities of Cu MOF nanozyme.

### 2.6 Improvement of Hypoxia and Inhibition of Synovitis in CIOA Mice After Cu MOF Nanozyme Treatment

**Figure 6:**
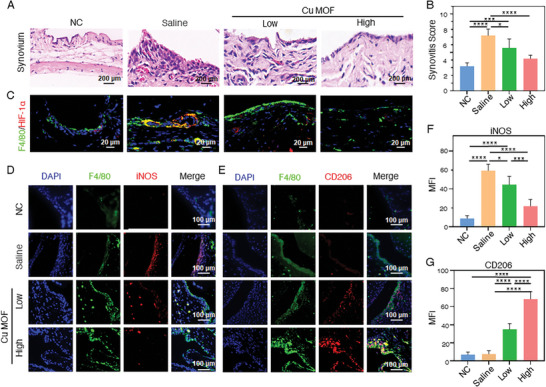
Improvement of hypoxia and inhibition of synovitis in CIOA mice after Cu MOF nanozyme treatment. A) H&E staining and B) synovitis score of knee joint synovium in CIOA mice at the end of treatment; C) Immunofluorescence staining of HIF‐1*α* in synovial macrophages (blue (DAPI), green (F4/80), red (HIF‐1*α*)) at the end of treatment; D,E) Immunofluorescence staining of iNOS or CD206 in the knee joint synovium at the end of treatment (blue (DAPI), green (F4/80), red (iNOS or CD206)); F,G) Statistical analysis of Mean Fluorescence Intensity of iNOS or CD206 by immunofluorescence staining. One‐way ANOVA with Tukey's multiple comparisons test was used to compare the means of the values of groups. ^*^ indicate significant differences between groups, respectively (*p* < 0.05). MFI: Mean fluorescence intensity.

To further explore the mechanisms underlying the superior therapeutic effect of Cu MOF nanozyme on OA treatment, based on the previous results, we focused on the improvement of synovial lesions in mice, especially the hypoxic microenvironment of the synovium and the intensity of synovitis. Following treatment of the high‐concentration Cu MOF nanozyme, the thickness of synovial lining cells recovered significantly from seven to eight layers to approximately two to three layers, comparable to the NC group. Meanwhile, the inflammatory infiltration in the sublayer matrix decreased markedly, accompanied by a notable decrease in the number of synovial cells in the lower layer (**Figure** **6**A). The synovitis score of CIOA model mice also decreased distinctly after the high‐concentration Cu MOF nanozyme treatment, comparable to NC group (Figure 6B). The above results showed that the level of synovitis improved significantly after Cu MOF nanozyme treatment.

Based on the previous results, Cu MOF with comprehensive and robust enzyme‐like activities could scavenge ROS and alleviate hypoxia of synovial macrophages in vitro, thus promoting the polarization from the M1 subtype to the M2 subtype. In comparison to the NC group, the fluorescence intensity of HIF‐1*α* (Alexa Fluor 594) co‐localized with F4/80 (Alexa Fluor 488) significantly increased in the saline group, indicating an exacerbated level of synovial macrophage inflammation induced by hypoxia (Figure 6C). After different dose treatments of Cu MOF, the expression of HIF‐1*α* decreased prominently, demonstrating that Cu MOF nanozyme can also improve the hypoxic microenvironment in vivo. To further investigate the transformation of macrophages from pro‐inflammatory M1 to anti‐inflammatory M2 after Cu MOF treatments, each group of knee joint paraffin sections was stained with: F4/80 (Alexa Fluor 488), iNOS (Alexa Fluor 594) staining for M1 macrophages and F4/80 (Alexa Fluor 488), CD206 (Alexa Fluor 594) staining for M2 macrophages. Compared to the saline group, the fluorescence intensity and area of iNOS expression co‐located with F4/80 reduced significantly after Cu MOF treatments (Figure 6D,F). Meanwhile, the red fluorescence intensity and area of F4/80 and CD206 co‐localization in the Cu MOF treatment groups were much higher than that in saline group, even more robust than that of the NC group, indicating a remarkable enhancement of anti‐inflammatory immunity (Figure 6E,G). The overall results showed that Cu MOF nanozyme treatment could improve the oxidative and hypoxic microenvironments of the synovium and promote the polarization of synovial macrophages from M1 to M2 phenotype, resulting in the apparent improvement of arthritis symptoms in CIOA mice.

## 3 Conclusion

A Cu MOF nanozyme with a biomimetic metal–nitrogen active center is designed and applied for efficient and safe OA treatment for the first time. Due to the unique structure, Cu MOF nanozyme exhibits comprehensive and powerful antioxidant activities (SOD‐like, CAT‐like, and •OH scavenging activities) and negligible pro‐oxidant activities (OXD‐ and POD‐like activities), compared with the other two Cu‐based nanozymes (CuNC and CuO). Especially, the SOD‐like activity of Cu MOF is two orders of magnitude higher than that of the commonly used SOD simulations such as CeO_2_ and Prussian blue nanozymes. Density functional theory calculation further confirmed the origin of outstanding enzyme activities of Cu MOF. In vitro and in vivo results demonstrated that Cu MOF nanozyme showed an excellent scavenging ability on different types of ROS in macrophages and chondrocytes, and reduced the expression of HIF‐1*α* in macrophages by producing oxygen. As a result, the intra‐articular injection of Cu MOF nanozyme improved the oxidative and hypoxic microenvironments of OA, thus promoting the M2 polarization of macrophages, reducing the synovitis's intensity, and inhibiting the cartilage degeneration. Moreover, no histopathological abnormalities or damage to the mice organs were observed after Cu MOF treatment. The excellent biocompatibility and protective properties of Cu MOF nanozyme make it a valuable asset in treating ROS‐related ailments and a catalyst for advancing next‐generation nanozymes in clinical settings.

## Conflict of Interest

The authors declare no conflict of interest.

## Supporting information

Supporting Information

## Data Availability

The data that support the findings of this study are available from the corresponding author upon reasonable request.
