# Supplementary material for: Using Cu‐Based Metal–Organic Framework as a Comprehensive and Powerful Antioxidant Nanozyme for Efficient Osteoarthritis Treatment
Source: Adv Sci (Weinh). 2024 Jan 26;11(13):2307798. doi: 10.1002/advs.202307798 (PMC10987124; doi:10.1002/advs.202307798)
Supplement: Supplementary file 1 — Supporting Information [file ADVS-11-2307798-s001.pdf]

ADVANCED SCIENCE
Open Access

Supporting Information

for *Adv. Sci.*, DOI 10.1002/advs.202307798

Using Cu-Based Metal–Organic Framework as a Comprehensive and Powerful Antioxidant Nanozyme for Efficient Osteoarthritis Treatment

Bo Yu, Wei Sun, Juntao Lin, Chaoyu Fan, Chengxinqiao Wang, Zhisen Zhang, Yupeng Wang, Yonghua Tang, Youhui Lin* and Dongfang Zhou**

Supporting Information

Using Cu-based metal-organic framework as a comprehensive and powerful antioxidant nanozyme for efficient osteoarthritis treatment

Bo Yu[#], Wei Sun[#], Juntao Lin, Chaoyu Fan, Chengxinqiao Wang, Zhisen Zhang, Yupeng Wang, Yonghua Tang[*], Youhui Lin[*], Dongfang Zhou[*]

Dr. B. Yu, Dr. W. Sun, Dr. J. Lin, Dr C. Wang, Dr. Y. Wang, Prof. D. Zhou
Department of Orthopaedics and Traumatology & Department of Ultrasonic Diagnosis, Zhujiang Hospital; Key Laboratory of Mental Health of the Ministry of Education; NMPA Key Laboratory for Research and Evaluation of Drug Metabolism & Guangdong Provincial Key Laboratory of New Drug Screening, School of Pharmaceutical Sciences
Southern Medical University
Guangzhou 510515, P. R. China
E-mail: dfzhou@smu.edu.cn

Dr. C. Fan, Dr. Z. Zhang, Dr. Y. Tang, Prof. Y. Lin
Department of Physics, Research Institute for Biomimetics and Soft Matter, Fujian Provincial Key Laboratory for Soft Functional Materials Research
Xiamen University
Xiamen 361005, P. R. China
E-mail: 19820190154698@stu.xmu.edu.cn; linyouhui@xmu.edu.cn

[#]These authors contributed equally to this work

Supplementary experimental section

Materials

All chemicals used were of analytical grade and required no further treatment. 3,3,5,5-Tetramethylbenzidine (TMB) were supplied from Sigma Aldrich. Iodonitrotetrazolium chloride, terephthalic acid, $CuCl_2$•$2H_2O$, 4,4'-bipyridine were obtained from Macklin. The commercial copper oxide was purchased from Shanghai Bohr Chemical Reagent Co. Hydrogen peroxide (H_2O_2, 30 wt.%), NaH_2PO_4, and Na_2HPO_4 were purchased from Beijing Chemical Industry Group CO., LTD. Ultrapure water obtained from a Millipore system (18.2 MΩ; Millipore Co., USA) was used exclusively to prepare all aqueous solutions.

Instruments

Samples were imaged in a JEM1400 plus transmission electron microscope for toroidal dark-field imaging with an accelerating voltage of 100 kV. A JEM-2100F with an electron acceleration energy of 200 kV was used to record the high-resolution TEM and elemental mappings. PANalytical X-ray diffraction recorded the phase structure of the resultant catalysts using monochromatic Cu Kα radiation (λ = 1.5406 Å). XPS experiments were performed using an Escalab 250Xi (Thermo Fisher Scientific). The UV-Vis absorption spectroscopy (DRS) was carried out on a Lambda 750, PerkinElmer.

Calculations

First-principles calculations were performed within the density generalized function theory framework using the Vienna Ab initio Simulation Package (VASP).[1,2] The Perdew-Burke-Enzerhof (PBE) generalized[3] and projection-enhanced wave (PAW) methods [4] describe the exchange-correlation potential and ion-electron interactions. The adopted DFT-D3 method considered van der Waals (vdW) interactions. All geometry optimizations and energy calculations were performed in a plane-wave basis set up to an energy cutoff of 450 eV and the energy and force convergence criteria were 10^{-5} eV and 0.03 eV/Å, respectively. The Brillouin zone was sampled using a 3×3×1 Monkhorst-Pack k-point grid for CuNC 2×2×1 for Cu-X MOFs and 2×4×1 for CuO (111). The vacuum layer was set to 15 Å in the z-direction to avoid interactions between the adjacent single-molecule layers. Molecular species, such as H_2O, H_2O_2, and •OOH, were simulated in a 15 Å cubic lattice using the same cutoff parameters and convergence criterion as the slab.

The adsorption energies were calculated using the following equation:

$$E_{ads} = E_{slab+mol} - E_{slab} - E_{mol}$$

Eslab+mol denotes the total energy of the Cu-X MOFs slab with adsorbates on it, and Eslab and Emol are the energies of the isolated slab and adsorbate, respectively. The equation evaluated the charge density difference:

$$\Delta_{\rho} = \rho_{A+B} - \rho_A - \rho_B$$

where, ρX is the electronic charge density of X (X = A, B, A+B).

Synthesis of Cu MOF

Cu MOF was prepared by the self-assembly of $CuCl_2$•$2H_2O$ and 4,4'-bipyridine at room temperature. Typically, 2 mL of 4,4'-bipyridine (100 mM) was dissolved in ethanol and added dropwise to 18 mL of aqueous Cu^{2+} (5.6 mM), and the reaction was carried out for 2 h. The precipitate was collected by centrifugation, washed three times with ultrapure water, and dried overnight.

Synthesis of CuNC

Bulk carbon carriers were prepared by calcining dicyandiamide in a crucible at 550 ℃ (heating rate, 2.3 ℃ min^{-1}) in static air for 3 hours. The resulting sample was ground and then obtained by thermal exfoliation in static air at 500 ℃ (heating rate, 5 ℃ min^{-1}) for 5 h. $CuCl_2$•$2H_2O$ (174 mg) and carbon carriers (100 mg) were dispersed in an ethanol solution (20 mL), sonicated for 10 min, and then dried by rotary evaporation. The oven-dried powder (80 ℃) was then heated to 450 ℃ (heating rate, 5 ℃ min^{-1}) for 5 h in a stream of nitrogen. Using an ethanol solution, the resulting powder was thoroughly washed and then dried in an oven at 80 ℃. Finally, the powder was heated to 550 ℃ (heating rate, 2 ℃ min^{-1}) and maintained under the protection of a nitrogen stream for 5 h.

Measurement of SOD-like activity

The $•O_2^-$ scavenging can be indirectly detected by measuring the intensity of uptake of the products of iodonitrotetrazolium chloride (INT) oxidation by $•O_2^-$. First, xanthine (0.6 mM) and (0.6 mM) and xanthine oxidase (0.05 U mL^{-1}) were reacted for 1.5 min in phosphate buffer (0.1 M, pH 7.4) at room temperature to produce $•O_2^-$. Subsequently, different nanozymes were added and undisturbed for 1.5 min. Finally, the remaining $•O_2^-$ was detected with the INT probe undisturbed for 1.5 min. As determined by UV-Vis absorption spectroscopy, sulfur dioxide reduces INT to a red product with an absorption peak at 510 nm. The elimination of $•O_2^-$ was calculated by the following equation:

$$\text{Elimination}(\%) = [(A_1 - A_2) \div (A_1 - A_0)] \times 100\%$$

where, A_0 is the absorbance of INT, and A_1 and A_2 represent the absorbance at 510 nm without nanozymes and in the presence of nanozymes.

Measurement of CAT-like activity

The CAT-like activities of the different nanozymes were measured at room temperature using a dissolved oxygen meter (JPSJ-605, Leici. China). The nanozymes (20 μg mL^{-1}) and H_2O_2 were added sequentially to 10.0 mL phosphate buffer (0.1 M, pH 7.4). The CAT-like activity of the nanozymes was demonstrated by the catalytic decomposition of H_2O_2 to H_2O and O_2.

H_2O_2 could be decomposed into •OH and react with TA to produce fluorescent 2-hydroxyterephthalic acid with an excitation wavelength of 320 nm and an emission peak of 425 nm. In the presence of nanozymes, H_2O_2 decomposes into H_2O and O_2 and cannot produce fluorescent 2-hydroxy-terephthalic acid. Therefore, the elimination of H_2O_2 can be investigated by monitoring the fluorescent signal of 2-hydroxy-terephthalic acid. After phosphate buffer (0.1M, pH 7.4, 5 mL) containing H_2O_2 (100 mM) and nanozymes (1 mg mL^{-1}, 100 μL) was vortexed hard and incubated at room temperature for 6 h. TA (0.5 mM) in DMF was added. The fluorescence of this mixture was then measured.

Hydroxyl radical scavenging activity of nanozymes

The •OH radical was generated by the Fenton reaction of 0.3×10^{-3} mM H_2O_2 and 0.5×10^{-3} mM $FeSO_4$ for 1 min. Then, 100 μL of nanozymes (0.1 mg mL^{-1}) were added to the solution and incubated for another 1 min to eliminate •OH. Finally, 3×10^{-4} M salicylic acid (SA) was introduced to detect residual •OH. SA was oxidized by •OH to 2, 3-dihydroxybenzoic acid with a purple color product having an absorption peak at 520 nm. The residual •OH was quantified by UV-vis absorption spectrometry. The following equation calculated the removal of •OH:

$$\text{Elimination}(\%) = [(A_1 - A_2) \div (A_1 - A_0)] \times 100\%$$

where, A_0 is the absorbance of SA and $A_{1,}$ and A_2 is the absorbance of 2,3-dihydroxybenzoic acid at 520 nm in the absence and presence of nanozymes.

Measurement of OXD-like activity of nanozymes

The OXD-like activity of nanozymes was examined by O_2-mediated oxidation of TMB. Unless otherwise stated, the reaction was carried out in 1 mL of PBS buffer (0.1 M, pH 5), 10 μL TMB (50 mM), and 50 μL nanozymes (1 mg mL^{-1}). The absorption spectra of the reaction solutions were recorded after 2 min of reaction at room temperature.

Measurement of POD-like activity of nanozymes

The exact process was followed for the OXD-like activity test, except that 10 μL of H_2O_2 (10M) was added at the same time as the TMB.

Preparation of mice primary chondrocytes

6-7 days of milk mice purchased from the Laboratory Animal Center of Southern Medical University. The femur and tibial plateau were freed. Carefully isolate the cartilage tissue at the femoral head and tibial platform of lactating mice with the assistance of the visual field of the asana microscope; pay attention to controlling the time for separating cartilage tissue. After isolating the cartilage at the femoral head and tibial plateau of lactating rats, the articular cartilage tissue was soaked in sterile PBS buffer (Gibco, USA) containing 10% penicillin-streptomycin solution for more than 40 min. (4.5 mL PBS+500 μL penicillin-streptomycin solution(Gibco, USA)).The tissue blocks soaked for more than 40 min were transferred into the cartilage tissue soaked in a Petri dish, and the chopped cartilage tissue was digested by type II collagenase(Sigma, Germany) and digested in a 5% CO_2 incubator at 37 ℃ for 6 h. Add a 15 mL centrifuge tube, 3 times the volume of DMEM/F12 medium containing 10% FBS, and centrifuge at room temperature 800 rpm for 5 minutes to obtain cell pellets. Wash the pellet with 3 mL of PBS and resuspend with 5 mL of DMEM/F12 medium supplemented with 10% FBS.

Cellular uptake and cell viability test

RhoB-labeled Cu MOF nanozymes were used to identify cellular uptake. Raw264.7 (Purchased from ATCC) and mice primary chondrocytes were cultured with RhoB-labeled Cu MOF nanozymes (80 μg mL^{-1}) for 24 h, and DAPI (Thermo Fisher Scientific, USA) was used for nuclei staining, followed by fluorescence microscopy analysis.

Cell viability was assessed using Cell-Counting-Kit-8 (CCK-8) (MCE, USA). After raw 264.7 was incubated with Cu MOF nanozyme (0-100 μg mL^{-1}) for 24 h, CCK-8 solution was added, and the absorbance was detected at 450 nm using a microplate reader (Tecan Trading AG, Switzerland).

Intracellular ROS assay

After pre-incubation with Cu MOF nanozymes (80 and 8 μg mL^{-1}) for 1 h, Raw264.7, and chondrocyte were incubated with or without 100 ng mL LPS/IFN-γ for 24 h, and IL-1β (10 ng mL^{-1}) for 24 h. The DCFH-DA reagent (Sigma, Germany) was used to co-culture with cells for 30 min. The medium was centrifuged to obtain the supernatant and the extracellular ROS.

The intracellular ROS content in raw264.7 was detected using flow cytometry (Beckman Coulter, Fullerton, CA, USA).

Intracellular •O_2^- , H_2O_2 and •OH detection

The cell treatment method was the same as the previous treatment. In the presence of HRP, ADHP reacts with H_2O_2 in a 1:1 stoichiometry to produce highly fluorescent Resorufin. The Resorufin product can be easily read by a fluorescence microplate reader with an excitation of 530-560 nm and an emission of 590 nm. Fluorescence values are proportional to the H_2O_2 or peroxidase levels within the samples. Cell Meter Mitochondrial Hydroxyl Radical Detection Kit (AAT Bioquest, USA) was used to detect hydroxyl radical production in cells. Di hydroethidine (Sigma, Germany) detects intracellular superoxide anion levels. Mix the cells and staining solution in a 1:1 ratio and incubate for 1 h at 37 ℃.

qPCR and Western Blotting analysis

After pre-incubation with Cu MOF nanozymes (80 and 8 μg mL^{-1}) for 24 h, Raw264.7, and chondrocyte were incubated with or without 100 ng mL LPS/IFN-γ and IL-1β (10 ng mL^{-1}) for 24 h. Raw264.7 and chondrocytes were washed using PBS after pre-treatment with Cu MOF nanozymes (80 μg mL^{-1}) for 1 h. After adding fresh DMEM with 100 ng mL^{-1} LPS, Raw264.7 was incubated under hypoxic or normal conditions (37 ℃, 5% CO_2) for 6 h. Mice chondrocytes were incubated with fresh DMEM/F12 (Gibco) supplemented with 10 ng/mL IL-1β (Peprotech, USA) for 24 h. Total RNA was extracted using TRIzol (Invitrogen, USA), and proteins were extracted using RIPA (Beyotime, China). Table S1 (Supporting Information) (IgeBio, Guangzhou, China) lists the primers used for qPCR. The concentration of total RNA was detected using a NanoDrop spectrometer (ND-2000, Nanodrop Technologies, Wilmington, DE). A TB Green Premix Ex TaqTM II Kit (Takara, Japan) was used for RT-PCR on a Step OnePlus Real-Time PCR System (Thermo Fisher, USA). The sequence of primers can be found in the supplementary information. Protein concentration was detected using a BCA Protein Assay Kit (Beyotime, China). Primary anti-collagen II, anti-MMP13, anti-MMP3, anti-CD86, anti-Arg-1, anti-HIF-1α, and anti-β-actin antibodies were purchased from Abcam.

Flow cytometry analysis

The cell treatment method is the same as the previous treatment. Ice-cold samples containing 10^5-10^6 cells per 100 μL were prepared. Permeabilize: add 100 μL permeabilizing agent to each tube. Incubate for 15 min at 4 ℃. Incubate with 0.1-10μL mL^{-1} fluorochrome-

conjugated primary antibody for 45 min at 4 ℃ in the dark. Resuspend in ice-cold PBS, 10% FBS, and 1% sodium azide to a final volume of 0.5-1 mL. Store at 4°C until analysis (within 24 h).

Immunofluorescence of raw264.7 and chondrocyte

After pre-incubation with Cu MOF nanozymes (80 and 8 μg mL^{-1}) for 24 h, raw264.7 were incubated with or without 100 ng mL^{-1} LPS/IFN-γ for 24 h. The sample was completely covered with 5% blank goat serum, and the slices should be placed in a wet box and incubated in a constant temperature and humidity incubator at 37 ℃ for 30 min; according to the instructions, the antibody is diluted in the antibody diluent; absorb the sealing solution, add the diluted first antibody, incubate overnight at 4 ℃; put the sample at room temperature, rewarm 15 min, remove the antibody working solution, wash once with buffer TBST for 5 min. Wash 3 times with buffer TBS for 5 minutes each time; dilute the antibody in antibody diluent as required by the instructions; avoid light at room temperature for 1 hour; remove secondary antibody working solution and wash with buffer TBST for 5 minutes; wash with buffer TBS for 3 times for 5 min each time; add DAPI working solution to the sample, avoid light, room temperature, incubate for 10 minutes; remove DAPI working solution, wash 1 time with buffer TBST for 5 min. Wash with buffer TBS for 3 times for 5 min each time. After adding anti-fluorescence attenuation sealing tablets, the images were observed and collected under the fluorescence microscope.

CIOA mice model

All animal work was performed in accordance with Laboratory animal Guideline for ethical review of animal welfare (the National Standard of the People’s Republic of China, GB/T 35892-2018). Five male adult mice of C57BL6/J about 8 weeks old were randomly divided into two groups (NC, sham) representing the blank group, sham operation group (control group), stable feeding for 1 week, respectively on the first and third days (recorded as d1, d3) after anesthesia, 10 μL of normal saline was injected into the knee joint cavity of mice in one NC group as a blank control, and 1U collagenase VII. was injected into the joint excavation of the right knee joint in the sham group d1 and d3, respectively. Antibiotics were injected intraperitoneally to prevent infection after surgery.

Efficacy of Cu MOF nanozyme *in vivo*

3 days after CIOA induction, mice were divided into four groups. Intra-articular saline injections of Cu MOF nanozymes were given every 7 days in each group. Day 0 was

designated as the first day of treatment. At 8 weeks, all mice were euthanized via anesthesia overdose. The knee joints were collected for evaluation. The knees were homogenized in PBS (1 mL) before being centrifuged (10000 ×g) for 15 min at 4 ℃. The remaining knee joint samples were fixed by immersion in paraformaldehyde (4%) for 1 week and then placed in ethylenediaminetetraacetic acid dihydrate (10%) reagent for decalcification for 2 weeks. After embedding the specimens in paraffin, they were cut into 7-μm thick sections. Then, the samples were stained with H&E to visualize morphology and safranin O to visualize glycosaminoglycan distribution. The sections were also stained with collagen II, iNOS, and CD206. All primary antibodies were purchased from Abcam.

Histological analysis

Histological changes in cartilage degeneration were assessed using the OARSI scoring system. The synovial inflammation scoring system was used to evaluate histological changes in the intensity of synovial inflammation. The independent researchers assessed the severity of osteoarthritis in each knee joint using the Osteoarthritis Injury Grading System of the International Association for the Study of Osteoarthritis (OARSI) and synovitis scores, taking the average as the final score for the knee joint.

Statistical analysis

All results are shown as the means ± standard deviation. The sample size (n) for each statistical analysis is shown in the figure legend. One-way ANOVA with a Tukey post hoc method was used to compare the mean values of groups. The difference was considered statistically significant when the p-value was less than 0.05. Statistical analyses were performed using GraphPad 9.

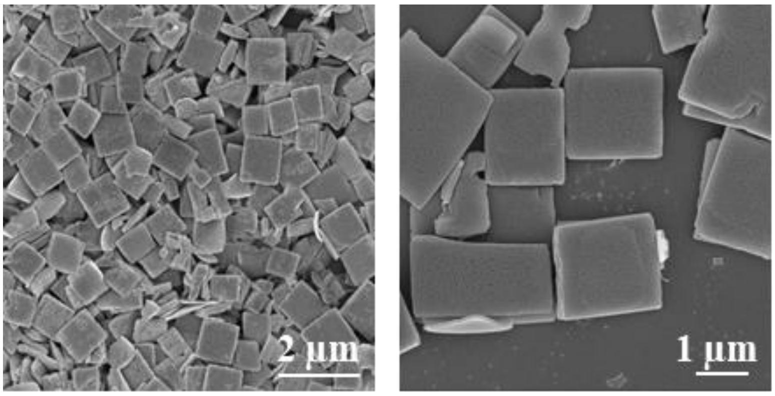

Figure S1 SEM images of Cu MOF.

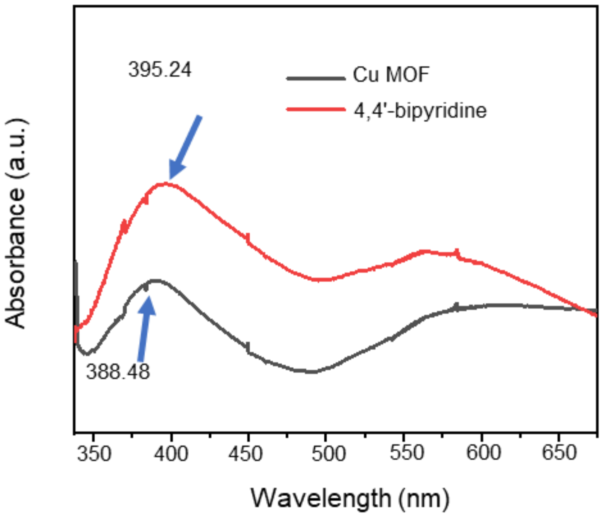

Figure S2 UV-vis absorption spectra of Cu MOF and 4,4'-bipyridine.

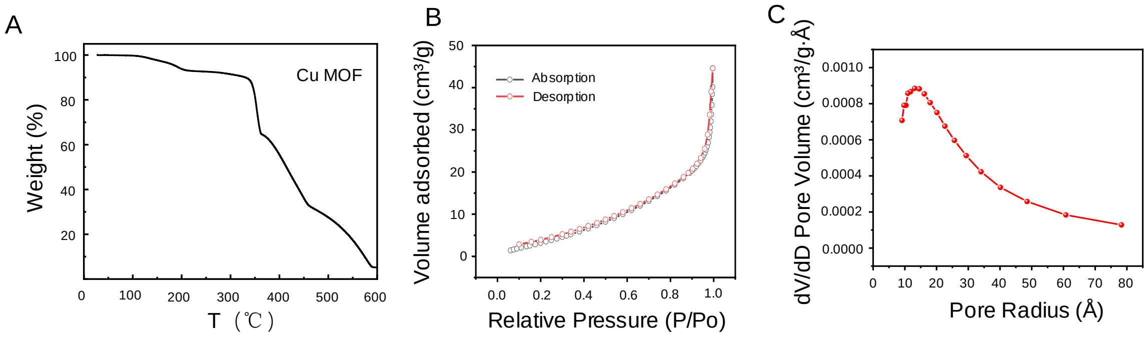

Figure S3 A) Thermogravimetry analysis patterns of Cu MOF. B) Adsorption-desorption isotherm of nitrogen and C) pore size distribution of Cu MOF.

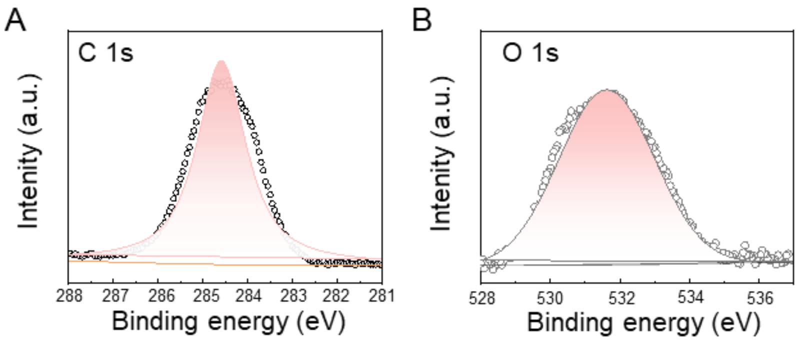

Figure S4 High-resolution XPS of Cu MOF. A) C 1s, B) O 1s.

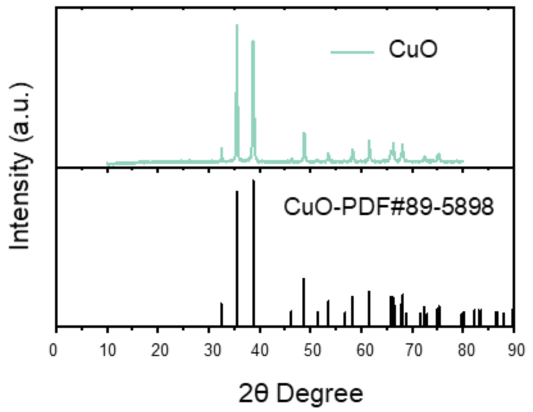

Figure S5 XRD pattern of CuO.

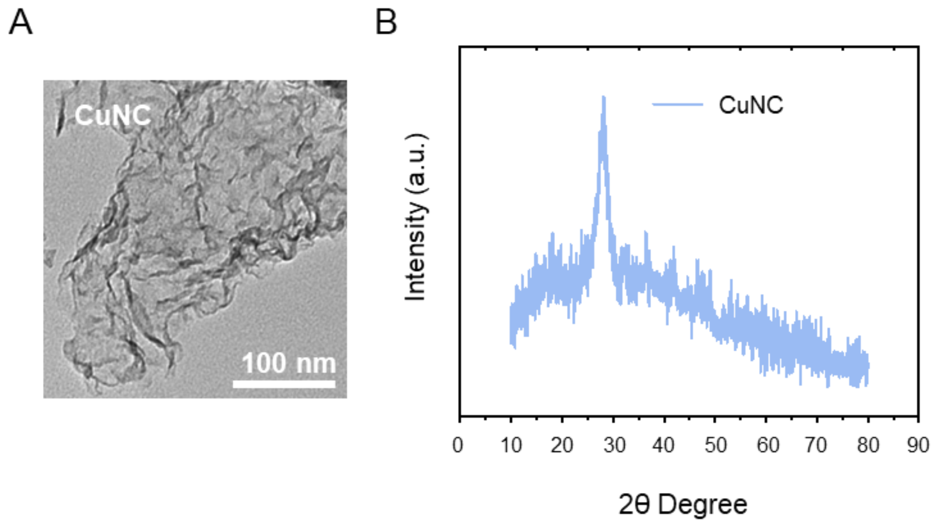

Figure S6 A) TEM image. B) XRD pattern for characterization of CuNC.

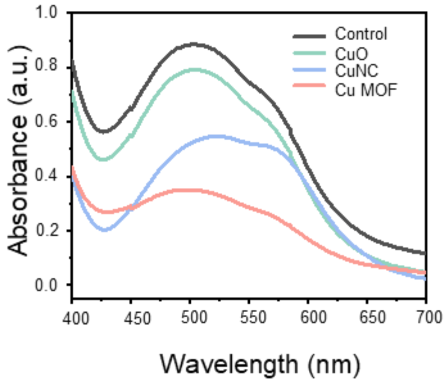

Figure S7 UV-vis absorption spectra of iodonitrotetrazolium chloride after reaction with xanthine and xanthine oxidase within different nanozymes.

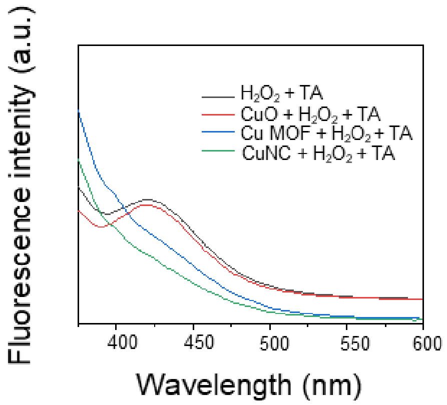

Figure S8 Fluorescent spectra of TA after reaction with H_2O_2 in the absence and presence of nanozymes.

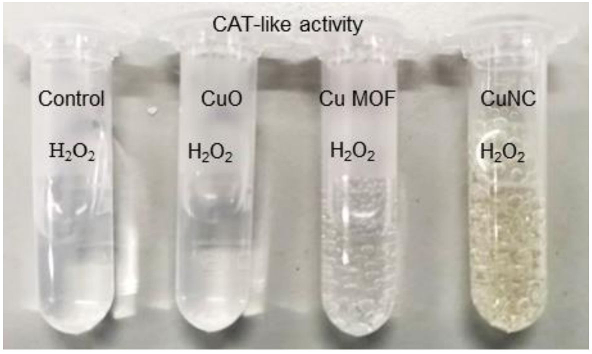

Figure S9 Photographs of different Cu-based nanozymes catalyzing the production of O_2 from H_2O_2.

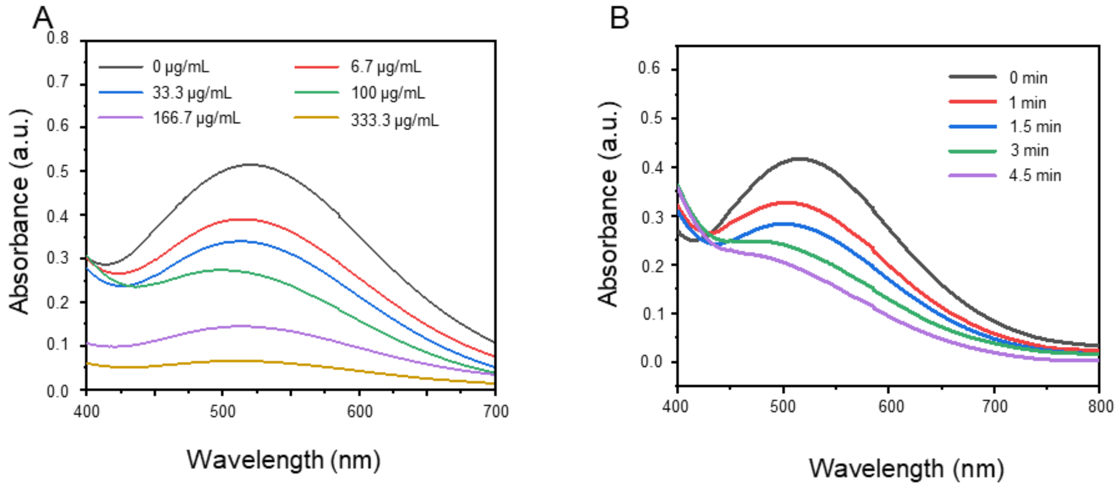

Figure S10 UV-vis absorption spectra at A) different concentrations and B) different Cu MOF treatment times of Cu MOF incubated with SA for determining •OH elimination.

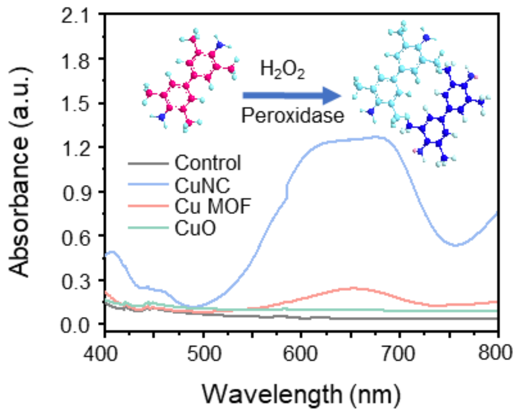

Figure S11 UV-vis absorption spectra of TMB and H_2O_2 after reaction with different Cu-based nanozymes.

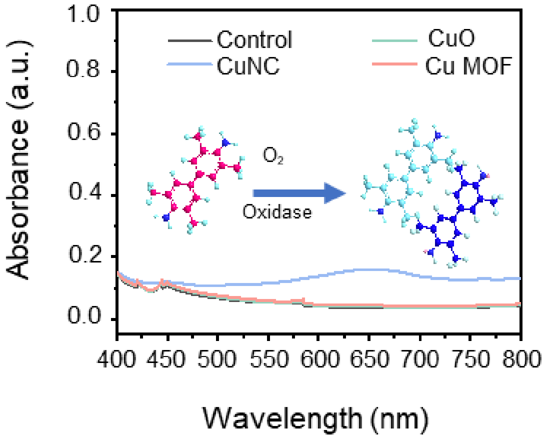

Figure S12 UV-vis absorption spectra of TMB after reaction with different Cu-based nanozymes.

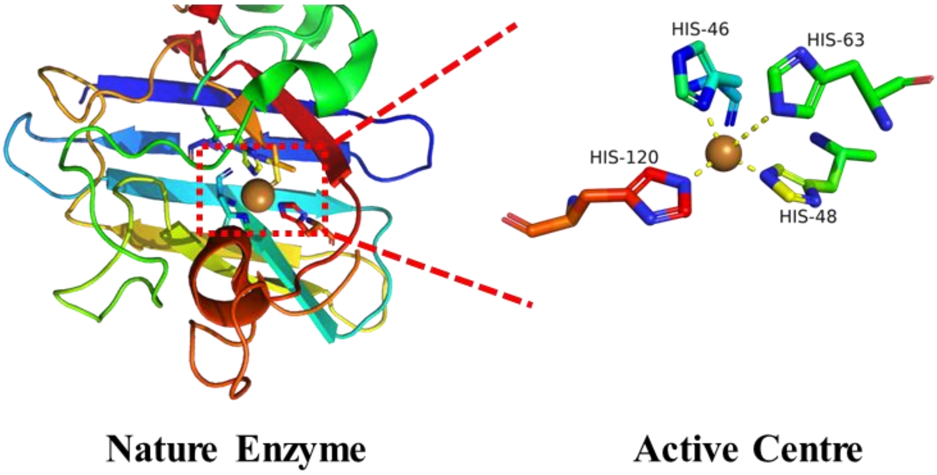

Figure S13 The Cu active center of natural Cu superoxide dismutase.

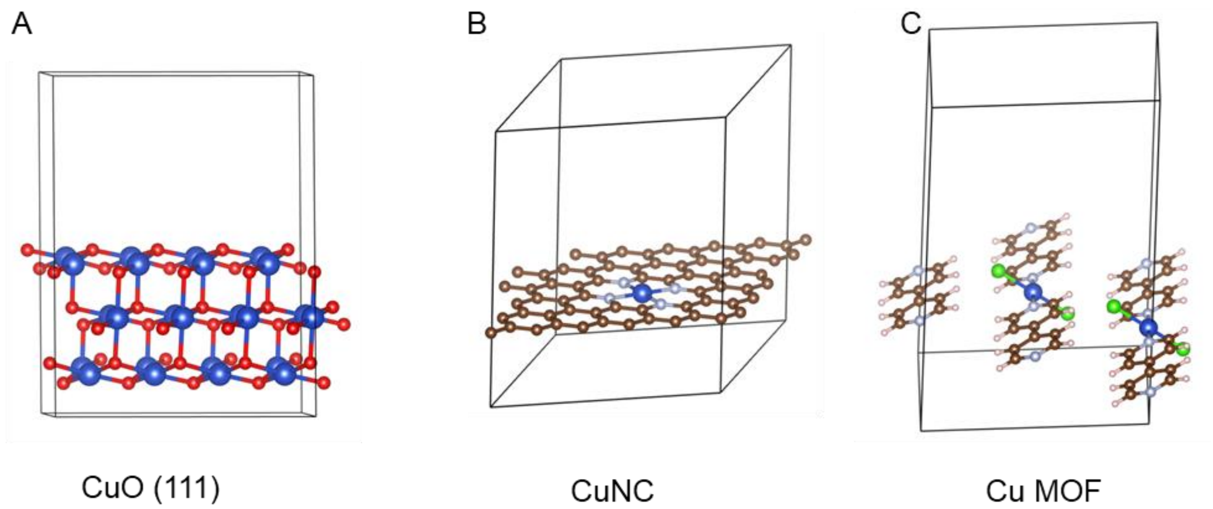

Figure S14 Computational models for different Cu-based nanozymes. A) CuO (111), B) CuNC, C) Cu MOF.

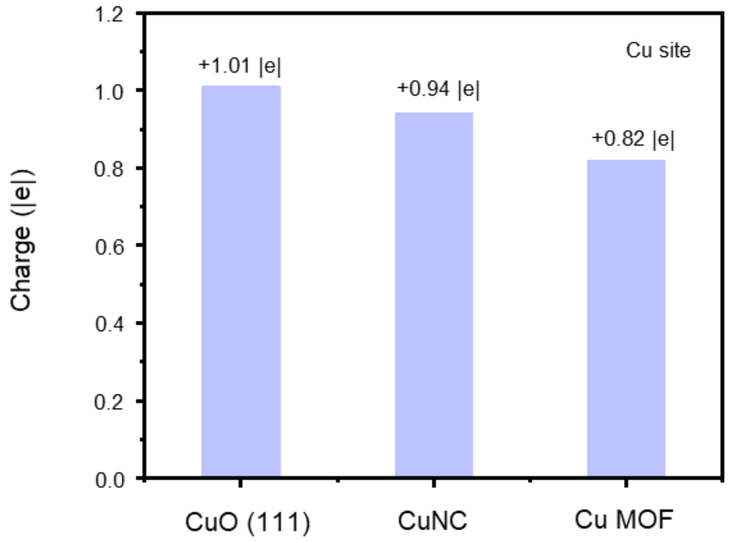

Figure S15 Bader charge of different Cu sites in CuO(111), CuNC, and Cu MOF.

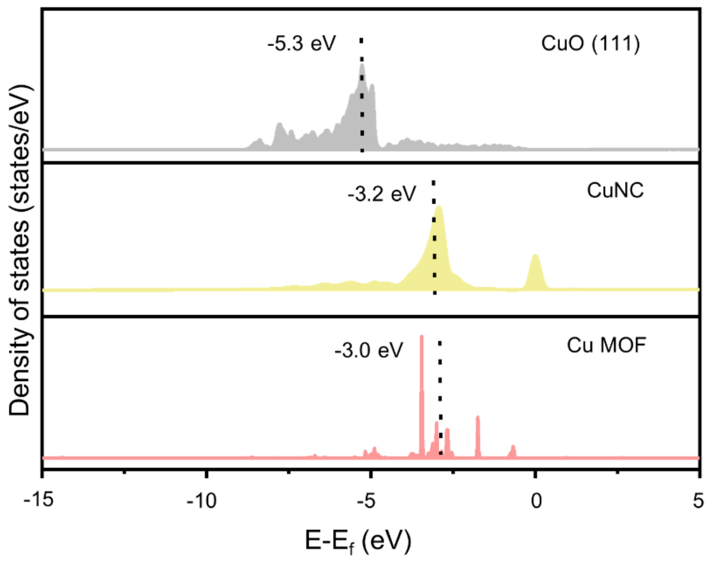

Figure S16 Projected density of states of d-bands of CuO (111), CuNC, and Cu MOF.

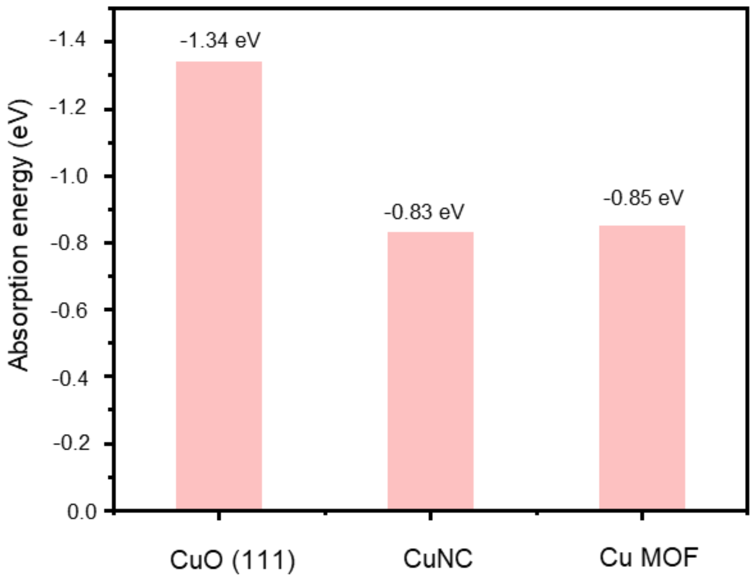

Figure S17 Absorption energy of •OOH molecules on the surface of different Cu-based nanozymes.

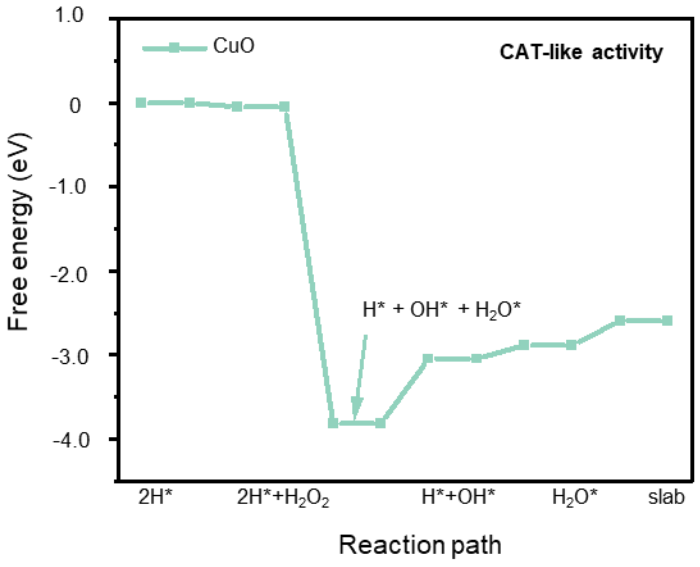

Figure S18 Subsequent pathways and intermediates of CAT-like activity of CuO (111).

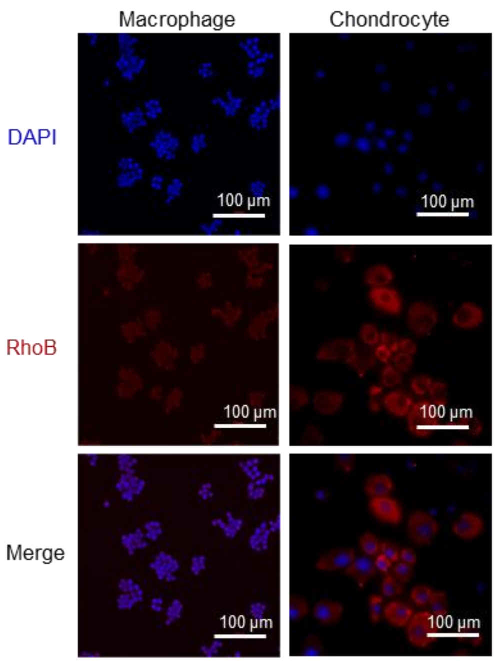

Figure S19 CLSM detected the uptake of RhoB-labeled Cu MOF in Raw264.7 cells. (blue: DAPI, red: RhoB-labeled Cu MOF)

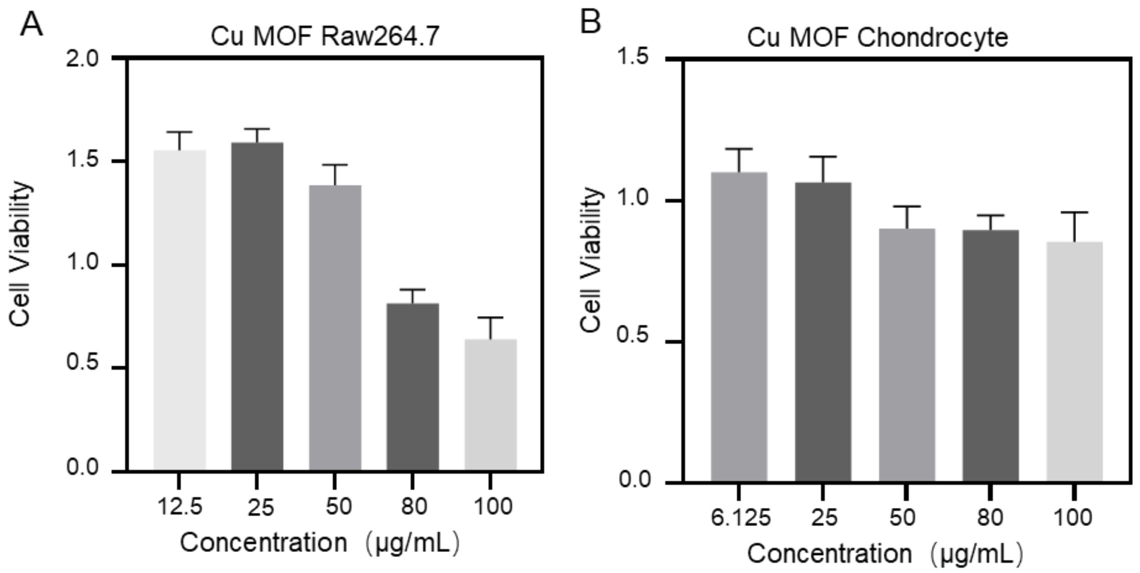

Figure S20 CCK-8 assay for testing cell viability of A) Raw264.7 cells and B) primary chondrocyte after incubation with Cu MOF at various concentrations for 48 h. Data are presented as the means ± SD (n=3).

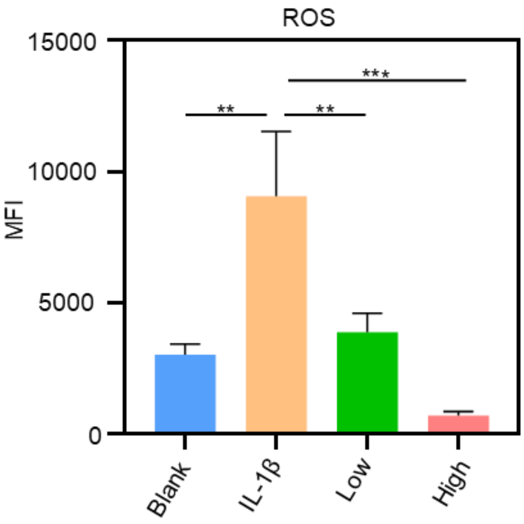

Figure S21 Flow cytometry analysis in Raw264.7 cells for ROS scavenging property induced by Cu MOF in vitro. After pre-incubation with Cu MOF nanozymes (80 μg mL^{-1}, 8 μg mL^{-1}) for 1 h, raw264.7 and chondrocyte were incubated with 100 ng mL LPS/IFN-γ for 24 h, and IL-1β (10 ng mL-1) for 24 h. * indicate significant differences between groups, respectively ($P < 0.05$). Data are presented as the means ± SD (n=3). One-way ANOVA with a Tukey post hoc method was used to compare the means of the values of groups.

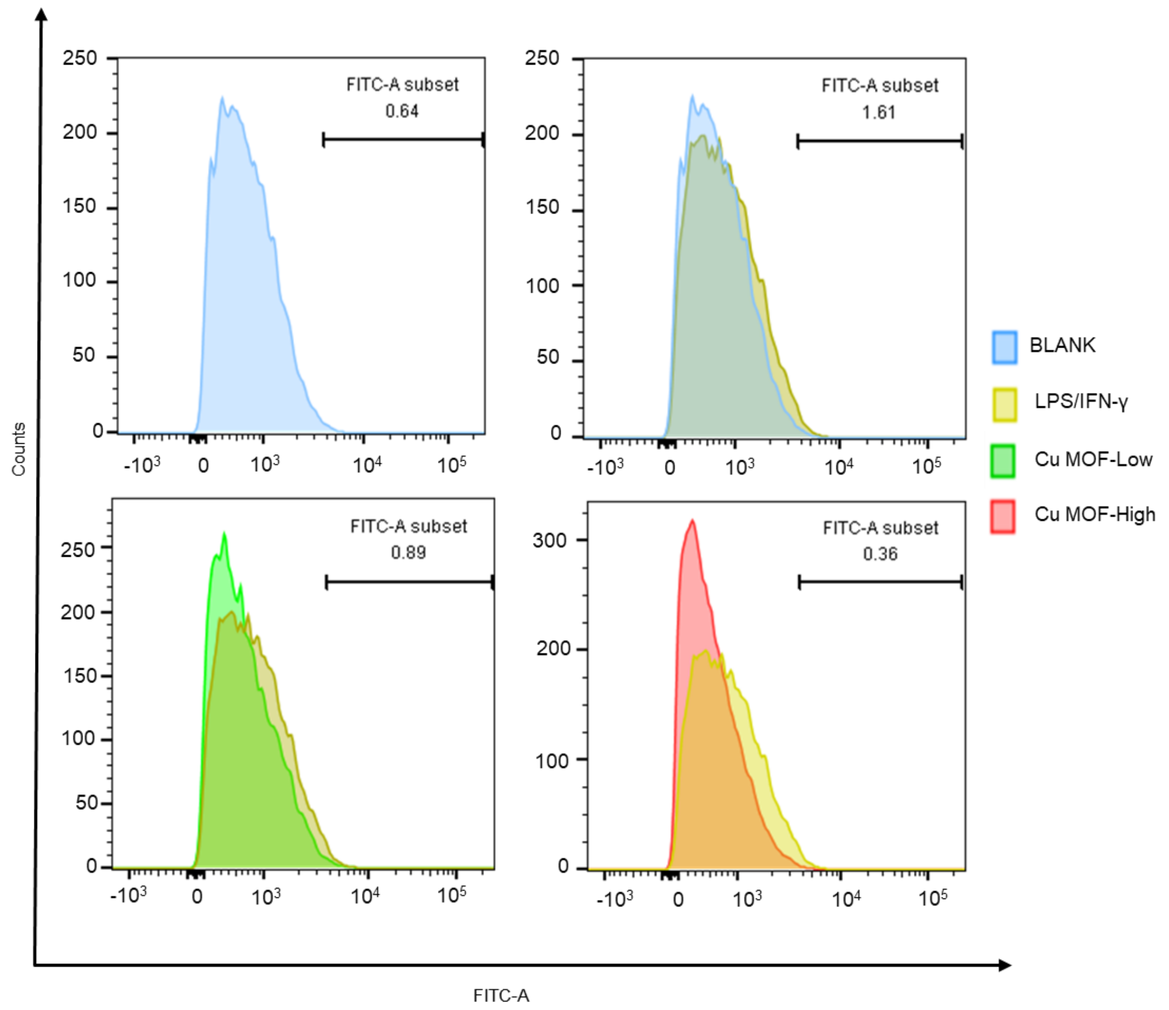

Figure S22 Flow cytometry analysis in Raw264.7 for ROS scavenging property induced by Cu MOF in vitro. The cell treatment method was the same as the Figure S21 treatment.

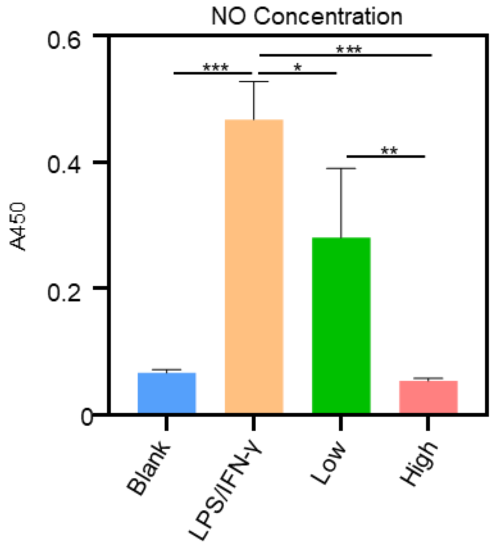

Figure S23 Microplate reader detected NO scavenging property induced by Cu MOF in vitro. The cell treatment method was the same as the Figure S22 treatment. * indicate significant differences between groups, respectively ($P < 0.05$). Data are presented as the means ± SD (n=3). One-way ANOVA with a Tukey post hoc method was used to compare the means of the values of groups.

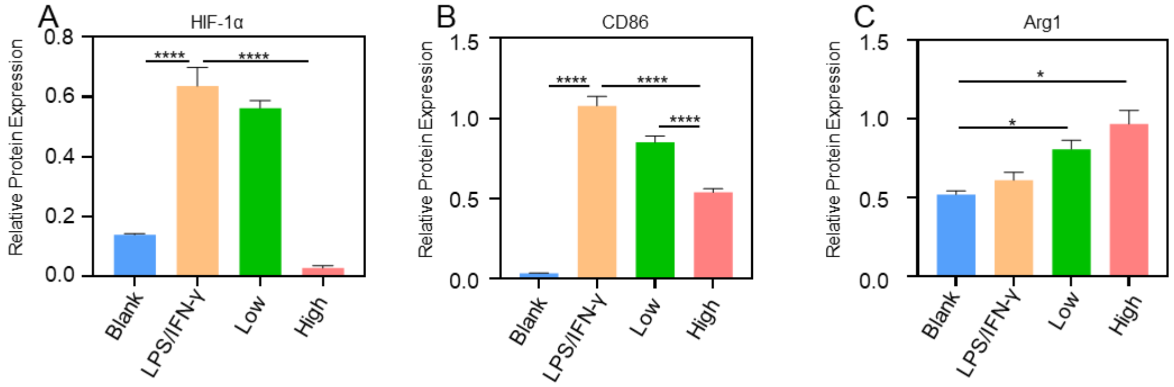

Figure S24 Western blotting analysis of A) HIF-1α, B) CD86, and C) Arg1 expression of macrophages under hypoxic cultures. Raw264.7 cells were pre-incubation with Cu MOF nanozyme (80 μg mL^{-1}, 8 μg mL^{-1}) for 24 h, then incubated with or without 100 ng mL^{-1} LPS/IFN-γ and IL-1β (10 ng mL^{-1}) for 24 h. The whole process was under hypoxic culture. Data are presented as the means ± SD (n=3). One-way ANOVA with a Tukey post hoc method was used to compare the means of the values of groups.

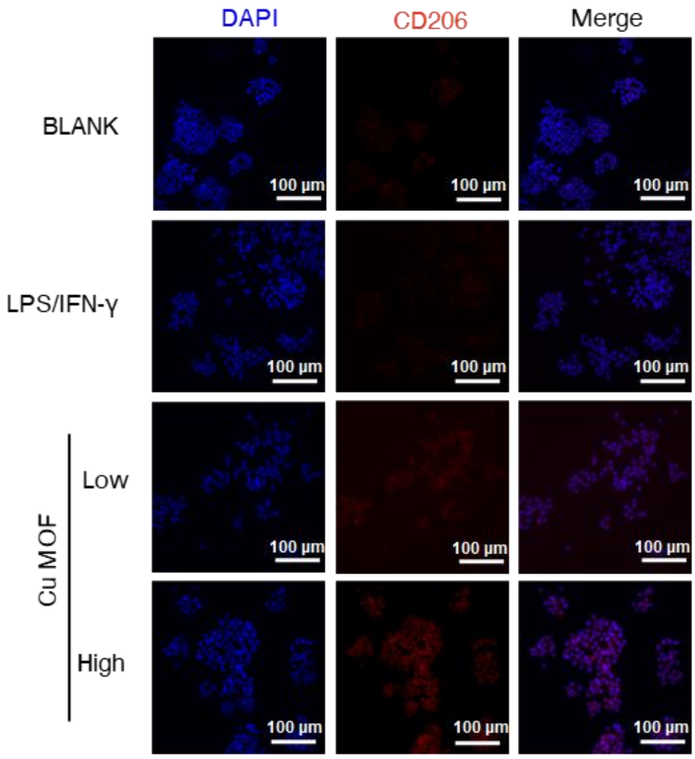

Figure S25 Immunofluorescence detection of CD206 expression in macrophages. Raw264.7 cells were pre-incubation with Cu MOF nanozyme (80 μg mL^{-1}, 8 μg mL^{-1}) for 24 h, then incubated with or without 100 ng mL LPS/IFN-γ for 24 h.

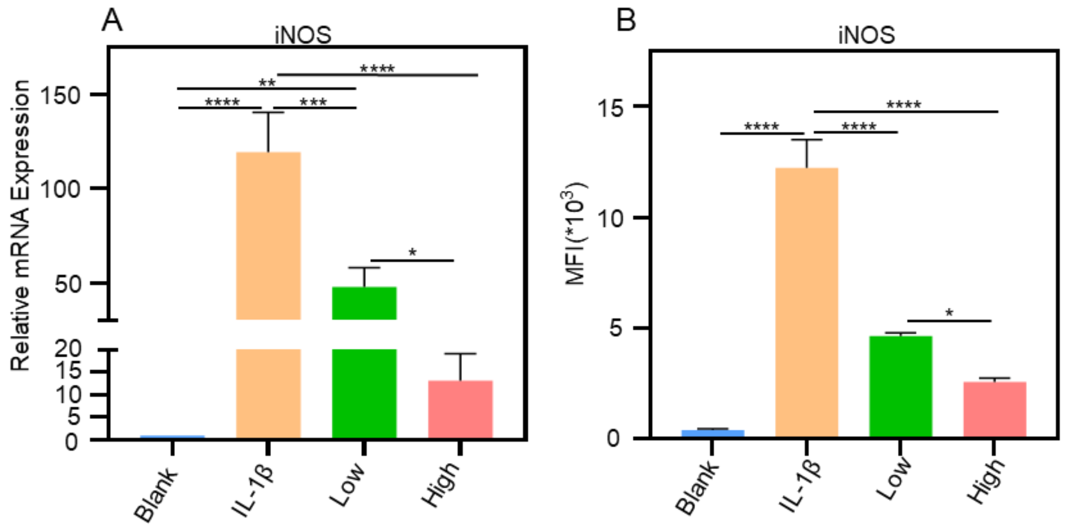

Figure S26 A) qPCR analysis and B) flow cytometry analysis of iNOS in macrophages after treatment with different doses of Cu MOF under inflammatory conditions (LPS/IFN-γ). The cell treatment method was the same as in Figure S25. * indicate significant differences between groups, respectively ($P < 0.05$). Data are presented as the means ± SD (n=3). One-way ANOVA with a Tukey post hoc method was used to compare the means of the values of groups.

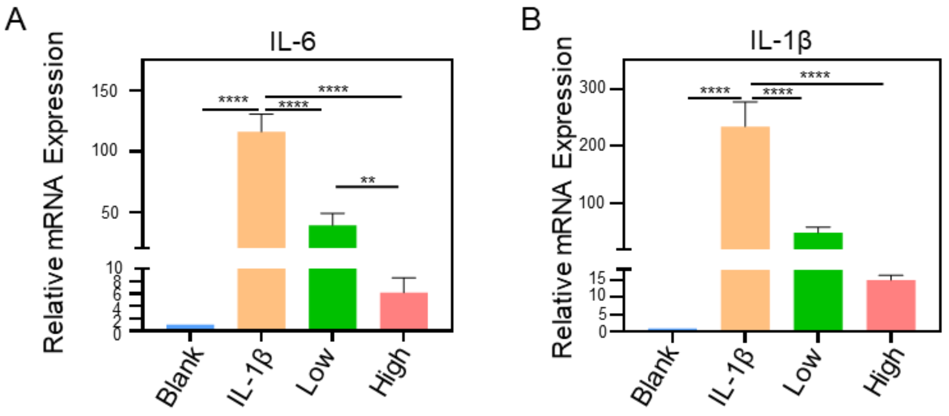

Figure S27 The qPCR analysis of A) IL-6 and B) IL-1β in macrophages after treatment with different doses of Cu MOF under inflammatory conditions (LPS/IFN-γ). The cell treatment method was the same as in Figure S26. * indicate significant differences between groups, respectively ($P < 0.05$). Data are presented as the means ± SD (n=3). One-way ANOVA with a Tukey post hoc method was used to compare the means of the values of groups.

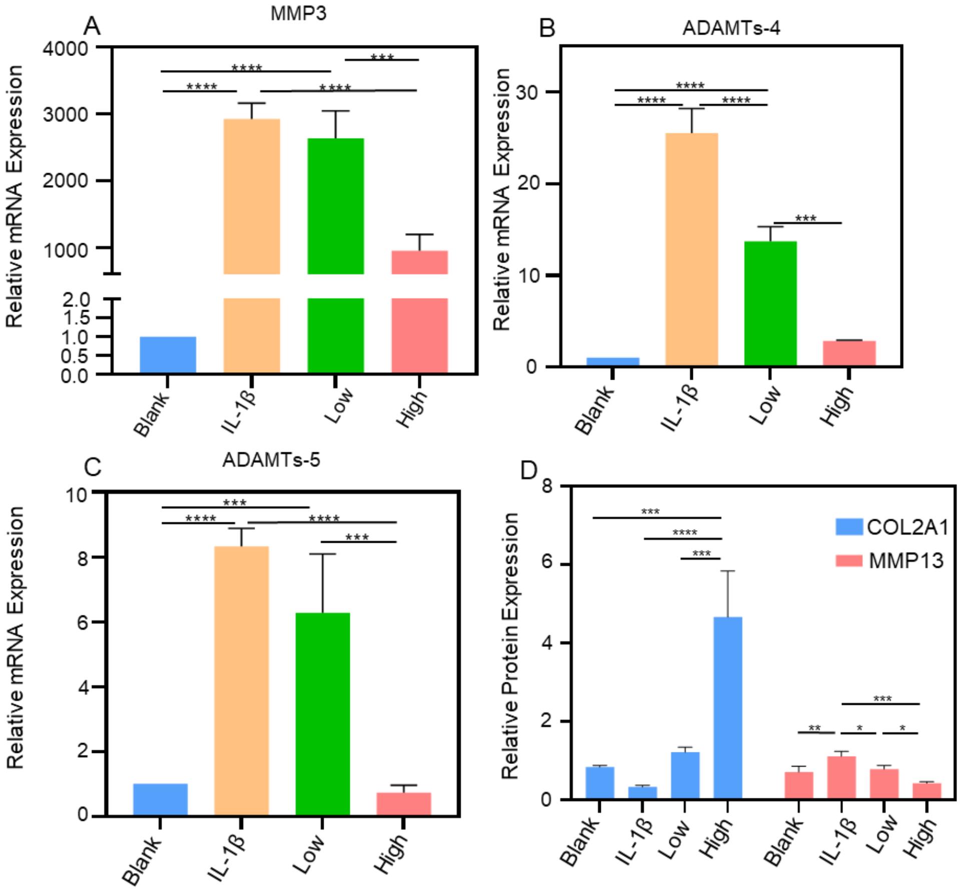

Figure S28 The qPCR analysis of cartilage ECM anabolic markers (MMP3, ADAMTs-4, and ADAMTs-5) from A) to C) in chondrocytes treated with different doses of Cu MOF under inflammatory conditions (IL-1β). D) Western Blotting analysis cartilage ECM anabolic markers (COL2A1, MMP-13) in chondrocytes treated with different doses of Cu MOF under inflammatory conditions (IL-1β). After pre-incubation with Cu MOF nanozyme (80 μg mL-1, 8 μg mL-1) for 24 h, chondrocytes were incubated with or without IL-1β (10 ng mL-1) for 24 h.* indicate significant differences compared with each other groups, respectively ($P < 0.05$). Data are presented as the means ± SD (n=3). One-way ANOVA with a Tukey post hoc method was used to compare the means of the values of groups.

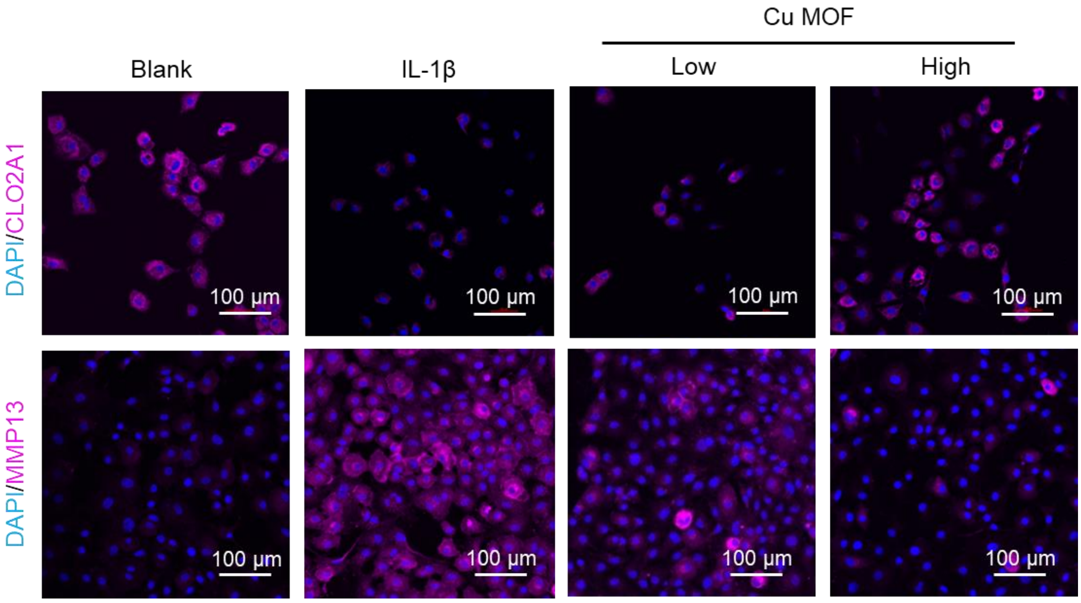

Figure S29 Immunofluorescence of cartilage ECM anabolic markers (COL2A1, MMP-13) in chondrocytes after treatment with different doses of Cu MOF under inflammatory conditions (IL-1β). The cell treatment method was the same as the Figure S28 treatment.

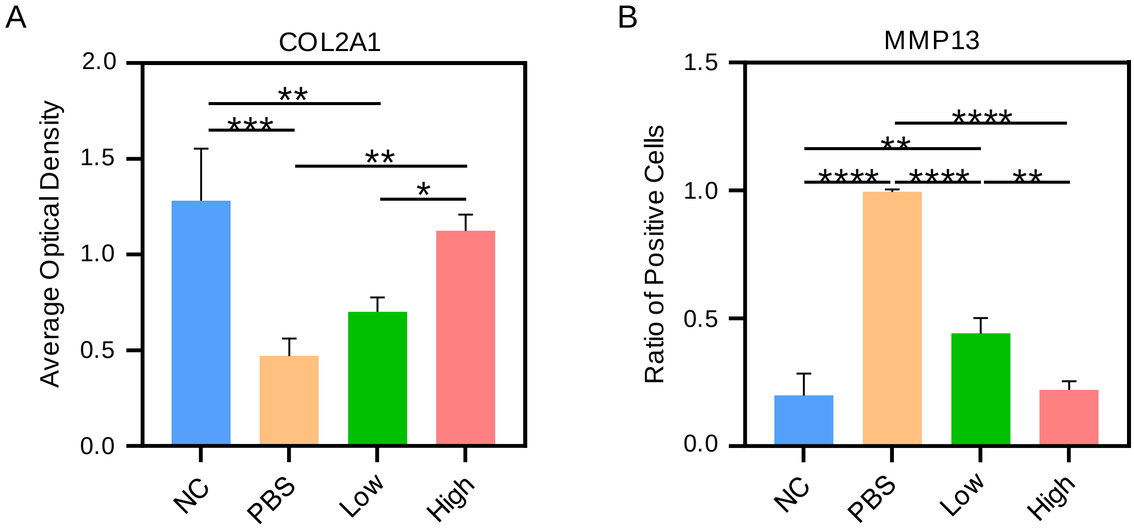

Figure S30 Semi-quantitative statistical analysis of immunohistochemical staining for tissue sections of knee articular tissues after different treatments.

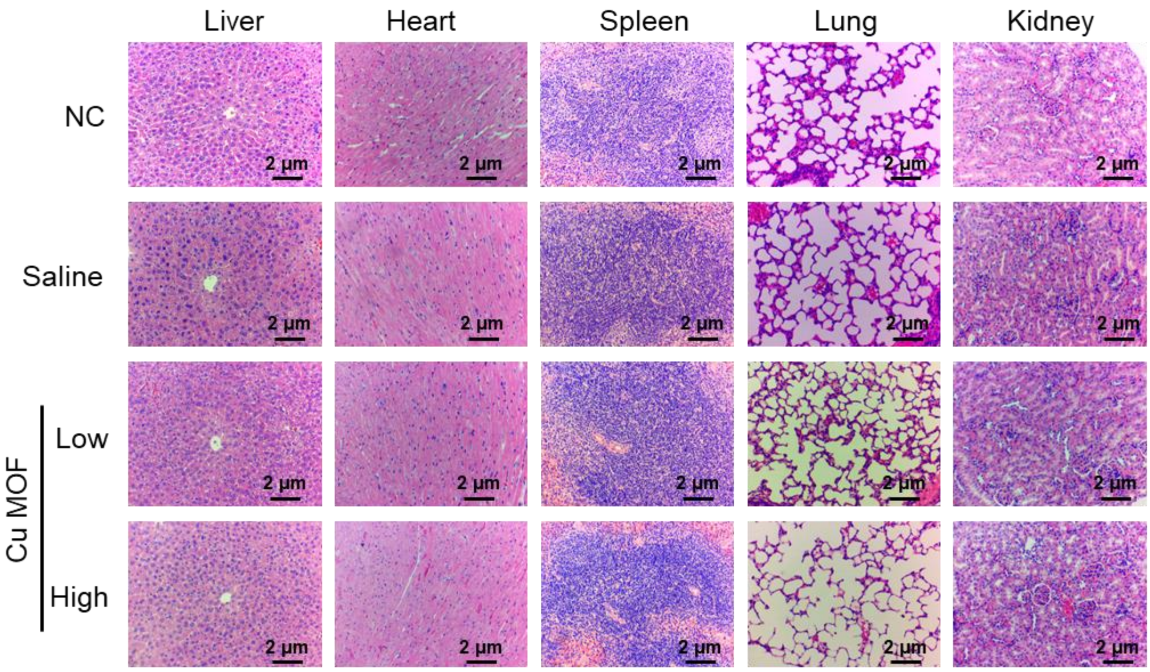

Figure S31 H&E staining images of tissue sections of various organs at the end of different treatments.

Table S1 The elemental content of the Cu MOF samples was obtained from XPS.

Elements	Atomic [%]
C	69.60
O	4.28
N	11.16
Cl	9.12
Cu	5.84

Table S2. Comparison of antioxidant activities of different nanozymes.

Samples	Removal efficiency			References
	SOD	CAT	·OH elimination	
CeO_2 NPs	~50.0% (20 μg mL^{-1})	~50.0% (20 μg mL^{-1})	-	[5]
PBzyme	46.4% (40 μg mL^{-1})	-	-	[6]
Pt@PCN-Mn-5	~65.0% (20 μg mL^{-1})	-	-	[7]
CuO	10.6% (0.1 μg mL^{-1})	6.8% (40 μg mL^{-1})	22.8% (100 μg mL^{-1})	This work
CuNC	40.3% (0.1 μg mL^{-1})	61.8% (40 μg mL^{-1})	~0% (100 μg mL^{-1})	This work
Cu MOF	70.0% (0.1 μg mL^{-1})	35.7% (40 μg mL^{-1})	50.8% (100 μg mL^{-1})	This work

Table S3. The primer sequences used in this study

Gene	Forward primer	Reverse primer
β-Actin	5’-CTAAGGCCAACCGTGAAAA-3’	5’-ACCAGAGGCATACAGGGACA-3’
MMP13	5’-GCCAGAACTTCCCAACCAT-3’	5’-TCAGAGCCCAGAATTTTCTCC-3’
MMP3	5’-TCCTGATGTTGGTGGCTTCA-3’	5’-TGTCTTGGCAAATCCGGTGTA-3’
ADAMTs 4	5’-CTTCCTGGACAATGGTTATG-3’	5’-GAAAAGTCGCTGGTAGATGGA-3’
ADAMTs 5	5’-GCCATTGTAATAACCCTGCAC-3’	5’-TCAGTCCCATCCGTAACCTTTG-3’
COL2A1	5’-CAAGAACAGCAACGAGTACCG-3’	5’-GTCACTGGTCAACTCCAGCAC-3’
IL-6	5’-GAGGATACCACTCCCAACAGAC-3’	5’-AAGTGCATCATCGTTGTTCATAC
IL-1β	5’-TGCCACCTTTTGACAGTGATG-3’	5’-AAGGTCCACGGGAAAGACAC-3’
Arg 1	5’-AGGGTCCACCCTGACCTATG -3’	5’- ACAGGTTGCCCATGCAGATT -3’

References

[1] G. Kresse, J. Furthmüller, *Comput. Mater. Sci.* **1996**, 6, 15.

[2] G. Kresse, J. Furthmüller, *Phys. Rev. B* **1996**, 54, 11169.

[3] J.P. Perdew, K. Burke, M. Ernzerhof, *Phys. Rev. Lett.* **1996**, 77, 3865.

[4] P.E. Blöchl, *Phys. Rev. B* **1994**, 50, 17953.

[5] J. Yao, Y. Cheng, M. Zhou, S. Zhao, S. Lin, X. Wang, J. Wu, S. Li, H. Wei, *Chem. Sci.* **2018**, 9, 2927.

[6] R. Hou, T. Lu, W. Gao, J. Shen, Z. Yu, D. Li, R. Zhang, Y. Zheng, X. Cai, *ACS Nano* **2022**, 16, 9559.

[7] Y. Liu, Y. Cheng, H. Zhang, M. Zhou, Y. Yu, S. Lin, B. Jiang, X. Zhao, L. Miao, H. Wei, *Sci. Adv.* **2020**, 6, eabb2695.
